# Reflections on *Menisporopsis*, *Multiguttulispora* and *Tainosphaeria* Using Molecular and Morphological Data

**DOI:** 10.3390/jof7060438

**Published:** 2021-05-31

**Authors:** Martina Réblová, Jana Nekvindová, Margarita Hernández-Restrepo

**Affiliations:** 1 Institute of Botany, Czech Academy of Sciences, 252 43 Průhonice, Czech Republic; 2 Department of Clinical Biochemistry and Diagnostics, University Hospital Hradec Králové, 500 05 Hradec Králové, Czech Republic; nekvindova@fnhk.cz; 3 Westerdijk Fungal Biodiversity Institute, 3508 AD Utrecht, The Netherlands; m.hernandez@wi.knaw.nl

**Keywords:** appendages, foliicolous, lignicolous, molecular systematics, phialidic conidiogenesis, taxonomic novelties

## Abstract

The genera *Menisporopsis*, *Multiguttulispora* and *Tainosphaeria* (Chaetosphaeriaceae) are saprobes inhabiting decaying plant material. This study is based on an integrated morpho-molecular characterisation to assess their generic concepts and explore phylogenetic relationships. *Menisporopsis* is revealed as polyphyletic, and species with 1-septate conidia and synnemata growing unilaterally along the seta are placed in the new segregate genus *Arcuatospora*. *Codinaea dimorpha* and *C. triseptata* are shown to be congeneric with *Multiguttulispora sympodialis*, the type species. Two new combinations are proposed: *M. sympodialis* is found conspecific with *M. dimorpha*. The *Tainosphaeria* complex is resolved into three genera. We found that the morphological separation of three groups within the genus is consistent with phylogenetic relationships. *Tainosphaeria* s. str. is accepted with five species. *Tainosphaeria aseptata* and *T. lunata* are transferred to the newly erected *Phialoturbella*, whereas *T. obclavata* is revealed as conspecific with *Phialogeniculata guadalcanalensis*, reducing it to a synonym. A new genus *Flectospora* is erected for a chloridium-like fungus nested in the *Tainosphaeria* clade. Based on molecular evidence, we show that asymmetrical, scolecosporous ascospores are a unique teleomorphic characteristic among family members. Therefore, we propose new combinations for *Chaetosphaeria hispida* in *Paragaeumannomyces* and *Ch. spinosa* in the new genus *Ericiosphaeria*, both exhibiting this rare morphotype.

## 1. Introduction

The present paper is concerned with the phylogeny and taxonomy of *Menisporopsis* [1], *Multiguttulispora* [2] and *Tainosphaeria* [3], classified in the Chaetosphaeriaceae. They occur mainly as dematiaceous hyphomycetous anamorphs, inhabiting leaf litter and decaying bark and wood. New morphological and DNA sequence data indicate that *Menisporopsis* and *Tainosphaeria* are associated with considerable morphological heterogeneity and genetic diversity. However, *Multiguttulispora*, though narrowly delimited, shows an undescribed affinity with known species.

The genus *Menisporopsis*, with *M. theobromae* as the type species, was established for fungi with pigmented, synnematous conidiophores growing around a central seta [1]. The conidiogenous cells are mono- or polyphialidic, producing aseptate, falcate, allantoid and fusiform hyaline conidia, with one to several setulae inserted at the ends or irregularly. Its members inhabit leaf litter, and occasionally decaying wood of various host plants in tropical and subtropical geographic areas [1,2,4,5,6,7]. The teleomorph is known only for *M. kobensis* [7]. Although most of the 13 species described in *Menisporopsis* [8] are congeneric with the type species, two species deviate. *Menisporopsis novae-zelandiae* has 1-septate conidia and synnemata are formed unilaterally along the seta [9], while *M. ludoviciana* is characterised by bacilliform conidia without setulae and single or fasciculate setae with a filiform extension [10,11]. Although *M. ludoviciana* was transferred to *Vermiculariopsiella* [12] based on morphological characters [13], the relationship of *M. novae-zelandiae* has not been evaluated.

Fernández and Huhndorf [3] erected *Tainosphaeria*, with *T. crassiparies* as the type species, for fungi resembling *Codinaea* [14]. *Tainosphaeria* lacks setae, the conidiophores are mononematous, pigmented, unbranched, and solitary with monophialidic conidiogenous cells and falcate, aseptate, hyaline conidia with a simple setula at each end. Ascomata are superficial and non-stromatic with unitunicate asci, persistent paraphyses and eight hyaline, fusiform, transversely septate ascospores. To date, seven species have been accepted in *Tainosphaeria* [8]. Admission of *T. aseptata*, *T. lunata* and *T. obclavata* introduced heterogeneity into *Tainosphaeria* [2,15], rendering the generic concept untenable. Their conidia lack setulae; they are falcate, lunate or oblong-clavate and aseptate, or even obclavate with a septum and distinctly geniculate conidiophores. Interestingly, the morphotype of *T. obclavata* has already been described as *Phialogeniculata guadalcanalensis* [16,17]. The emerging *Tainosphaeria* clade also includes other lineages with clearly distinct morphology, such as *Anacacumisporium* [18]. Preliminary phylogenetic analysis of ribosomal DNA sequences indicates a relationship between *Tainosphaeria* and the presumed chloridium-like anamorph of *Chaetosphaeria hispida* [19]. *Chaetosphaeria hispida* is a lignicolous species from Thailand with asymmetrical, scolecosporous, hyaline ascospores and dark, perithecial ascomata covered with short, opaque spines. The same traits also characterise *Ch. spinosa* [3] and are typical of *Paragaeumannomyces* [7,20].

*Multiguttulispora* [2] is a monotypic genus based on *M. sympodialis*, observed on decaying plant material in Thailand. It has mononematous, pigmented conidiophores, polyphialidic conidiogenous cells and transversely 3-septate, setulate conidia. However, the authors did not consider other species, such as *Codinaea dimorpha* [21] and *C. triseptata* [22], which share the same diagnostic characteristics. Because ex-type strains of both *Codinaea* species are not available, several non-type strains of *C. dimorpha* from Malaysia and Japan and *C. triseptata* from Cuba are included in this study to investigate the interspecific relationships of *Multiguttulispora*.

To evaluate the generic concepts of *Menisporopsis*, *Multiguttulispora*, *Tainosphaeria* and other morphologically similar genera, and explore their species boundaries and evolutionary relationships, we based our research on critical evaluation of morphotype data and phylogenetic analysis of rDNA sequences of the fresh material, ex-type strains and other isolates available in public collections.

## 2. Materials and Methods

### 2.1. Fungal Strains, Morphology, DNA Extraction and PCR Amplification

Specimens were collected in temperate forests in New Zealand and tropical monsoon forests in Thailand. Other samples were obtained from the National Museum (PRM, Prague, Czech Republic). Holotypes and specimens (as dried voucher specimens or dried cultures) were deposited at New Zealand Fungarium (PDD, Auckland, New Zealand), Westerdijk Fungal Biodiversity Institute (CBS, Utrecht, the Netherlands) and the Herbarium of the Institute of Botany (PRA, Průhonice, Czech Republic). Axenic cultures were derived from freshly collected material. Additional cultures were obtained from CBS and BCCM/MUCL Agro-food & Environmental Fungal Collection (MUCL, Université Catholique de Louvain, Louvain, Belgium). Representative strains and ex-type strains isolated from our collections were deposited at CBS and the International Collection of Microorganisms from Plants (ICMP, Auckland, New Zealand). Isolates, their sources and GenBank accession numbers of sequences generated in this study are listed in Table 1. Fungal novelties were registered in MycoBank.

Morphological characteristics were acquired from fungi growing on natural substrates and in culture. Ascomata, conidiophores and conidia from the natural substrates were rehydrated with tap water and examined with an Olympus SZX12 dissecting microscope (Olympus America, Inc., Melville, NY, USA). Sections of ascomatal wall, asci, ascospores and paraphyses, and conidiophores and conidia were mounted in 90% lactic acid, water or Melzer’s reagent. Measurements were taken in Melzer’s reagent. Means ± standard deviation (SD) based on a minimum of 20–25 measurements were given for sizes of asci, ascospores and conidia. Microscopic structures were examined using an Olympus BX51 compound microscope with differential interference contrast (DIC) and phase-contrast (PC) illumination. An Olympus DP70 camera operated by Imaging Software Cell^D (Olympus) were used to capture images of microscopic structures. Macroscopic images of colonies were documented using a Canon EOS 77D digital camera with Canon EF 100 mm f/2.8L Macro IS USM objective (Canon Europe Ltd., Middlesex, UK) with daylight spectrum 5500K 16W LED lights. All images were processed with Adobe Photoshop CS6 (Adobe Systems, San Jose, CA, USA).

Single and multiple ascospore and conidial isolates were obtained from fresh material with the aid of a single-spore isolator (Meopta, Přerov, Czech Republic) and incubated on water agar or Modified Leonian’s agar (MLA) [23] at a temperature of 20–25 °C. For comparison, strains were inoculated in triplicate on cornmeal dextrose agar (CMD) (Oxoid Limited, Hampshire, UK; 2% dextrose), MLA, oatmeal agar (OA) and potato-carrot agar (PCA) [24]. Descriptions of colonies were based on 4-week-old cultures grown in darkness at 22–23 °C. Strains were also inoculated on cornmeal agar (CMA) with sterile stems of *Urtica dioica* and synthetic nutrient agar (SNA) with pine needles [24] to induce sporulation.

Total genomic DNA was extracted from mycelium removed from 3–4-wk-old cultures grown on MLA using the DNeasy^®^ UltraClean^®^ Microbial Kit (Qiagen GmbH, Hilden, Germany) following the manufacturer’s protocol for filamentous fungi. All PCR amplifications were carried out in 25 μL volume reactions using a Q5 High Fidelity DNA polymerase kit (New England Biolabs Inc., Hitchin, UK) according to the manufacturer’s protocol.

Primers V9G/LR8 [25,26] were used for amplification of the internal transcribed spacer region (ITS1-5.8S-ITS2) (ITS) and the nuclear large subunit 28S rDNA gene (28S) (approximately 1800 base pairs at the 5′-end). PCR was carried out in a BioRad C1000 thermal cycler (Bio-Rad Laboratories Inc., Hercules, CA, USA) as follows: 98 °C for 30 s; 40 cycles of denaturation (98 °C for 10 s), annealing (62 °C for 30 s) and elongation (72 °C for 90 s) and a final extension step (72 °C for 5 min). Amplicons were purified from agarose gels using a NucleoSpin^®^ Gel and PCR Clean-up Kit (Macherey-Nagel GmbH & Co. KG, Dueren, Germany) following the manufacturer’s instructions, with an elution volume of 25 μL. The DNA concentration was assessed fluorimetrically using Quant-iT PicoGreen dsDNA Assay Kit and Qubit fluorometer (Invitrogen/Thermo Fisher Scientific Inc., Waltham, MA, USA) to assure the required sequencing concentrations adjusted for the length of amplicons/number of reads required.

The amplicons were sequenced in both directions using the PCR and nested primers: ITS5, ITS4, JS1, JS7, JS8 and LR7 [26,27,28,29]. Automated sequencing was carried out by Eurofins GATC Biotech Sequencing Service (Cologne, Germany) and Westerdijk Fungal Biodiversity Institute (Utrecht, the Netherlands). Raw sequence data were analysed using Sequencher v5.4.6 (Gene Codes Corp., Ann Arbor, MI, USA).

### 2.2. Alignments and Phylogenetic Analyses

Sequences of the ITS and 28S rDNA gene were analysed to assess relationships among species of *Menisporopsis*, *Multiguttulispora*, *Tainosphaeria* and similar fungi. The ITS and 28S rDNA sequences of the strain of *C. dimorpha* NBRC 33230 were retrieved from the NBRC online catalogue [30]. GenBank accession numbers for sequences retrieved from GenBank [31] and published in other studies [2,15,18,20,32,33,34,35,36,37,38,39,40,41,42,43,44,45,46,47,48,49] are listed in the supplementary file: Table S1.

Consensus secondary structure (2D) models for the ITS1 and ITS2 for members of the Chaetosphaeriaceae were built using the Ppfold program v3.0 [50]. The obtained 2D consensus models were further improved using the program Mfold [51] and RNAfold web server through the ViennaRNA Web Services [52,53] and adjusted manually if necessary, based on a comparison of homologous positions in the multiple sequence alignment. The predicted 2D RNA structures were obtained in a dot bracket notation and were visualized and drawn using the program VARNA: Visualization Applet for RNA [54].

The ITS-28S for newly sequenced taxa and those retrieved from GenBank and NBRC online catalogue were added to the combined ITS-28S alignment. Sequences were aligned manually in Bioedit v7.1.8 [55]. Consensus 2D structure models for the ITS1 and ITS2 were used to compare nucleotides at homologous positions (in helices and loops) in order to construct a reliable multiple sequence alignment. A predicted 2D model of the 28S of *Saccharomyces cerevisiae* [56] was used to improve the alignment of this gene. The models were highly consistent in all species. Initially, we considered applying Gblocks [57] to determine and remove putative ambiguous areas in the alignment. However, following a study of the Chaetosphaeriaceae by Réblová et al. [20], who tested performance of the alignment improved with 2D structure with and without Gblocks, we included the whole ITS-28S alignment except for 91 nucleotides (nt) at the 5′-end of 28S, because of the incompleteness in the majority of sequences.

The ITS and 28S datasets, for which we assumed rate heterogeneity, were evaluated using PartitionFinder2 [58], implemented in the CIPRES Science Gateway v3.3 [59,60], to find the best partitioning scheme for our datasets and to select best-fit models under corrected Akaike information criteria. The GTR+I+G model was selected for both partitions. The ITS and 28S data sets were concatenated, and the alignment (deposited in TreeBASE 28300) was subjected to phylogenetic analysis. The full dataset consisted of 2386 characters including gaps (ITS = 614 characters; 28S = 1772) and 1048 unique character sites (RAxML). *Tracylla aristata* and *T. eucalypti* (Tracyllales) were selected as outgroup taxa.

Phylogenetic reconstructions were performed using Bayesian Inference (BI) and Maximum Likelihood (ML) analyses through the CIPRES Science Gateway v.3.3. ML analysis was conducted with RAXML-HPC v8.2.12 [61] with a GTRCAT approximation. Nodal support was determined by non-parametric bootstrapping (BS) with 1000 replicates. BI analysis was performed in a likelihood framework as implemented in MrBayes v3.2.6 [62]. Two Bayesian searches were performed using default parameters. The B-MCMCMC analyses lasted until the average standard deviation of split frequencies was below 0.01 with trees saved every 1000 generations. The first 25% of saved trees, representing the burn-in phase of the analysis, were discarded. The remaining trees were used for calculating posterior probabilities (PP) of recovered branches. The BI and ML phylogenetic trees were compared visually for topological conflict among supported clades.

## 3. Results

### 3.1. Phylogenetic Analyses

**Figure 1:**
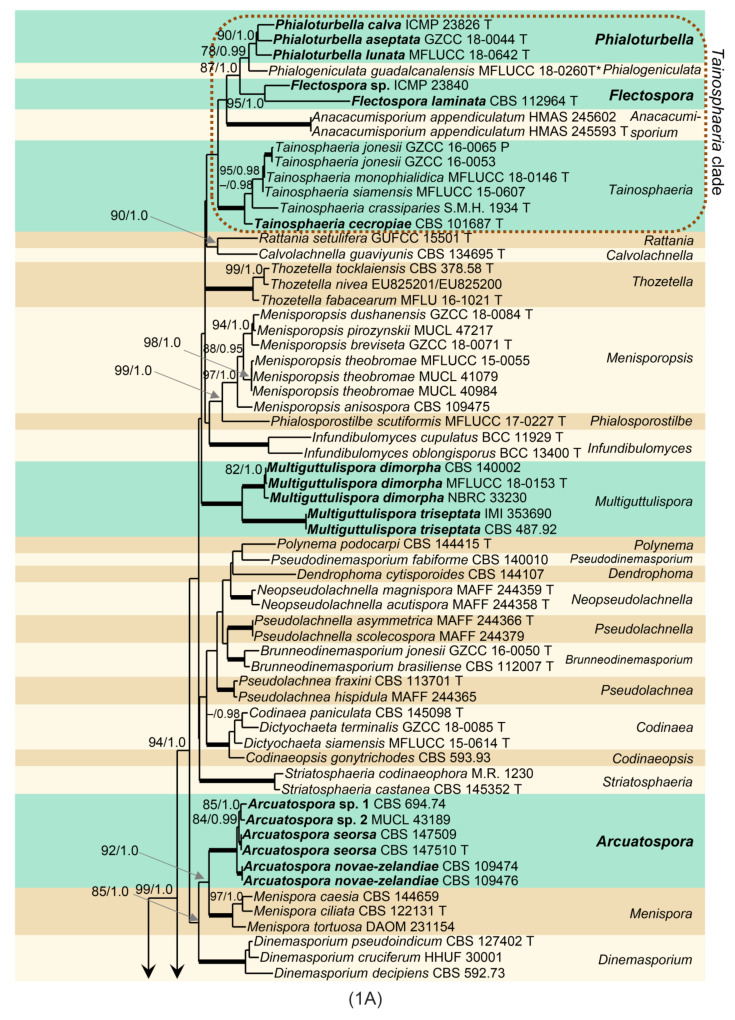

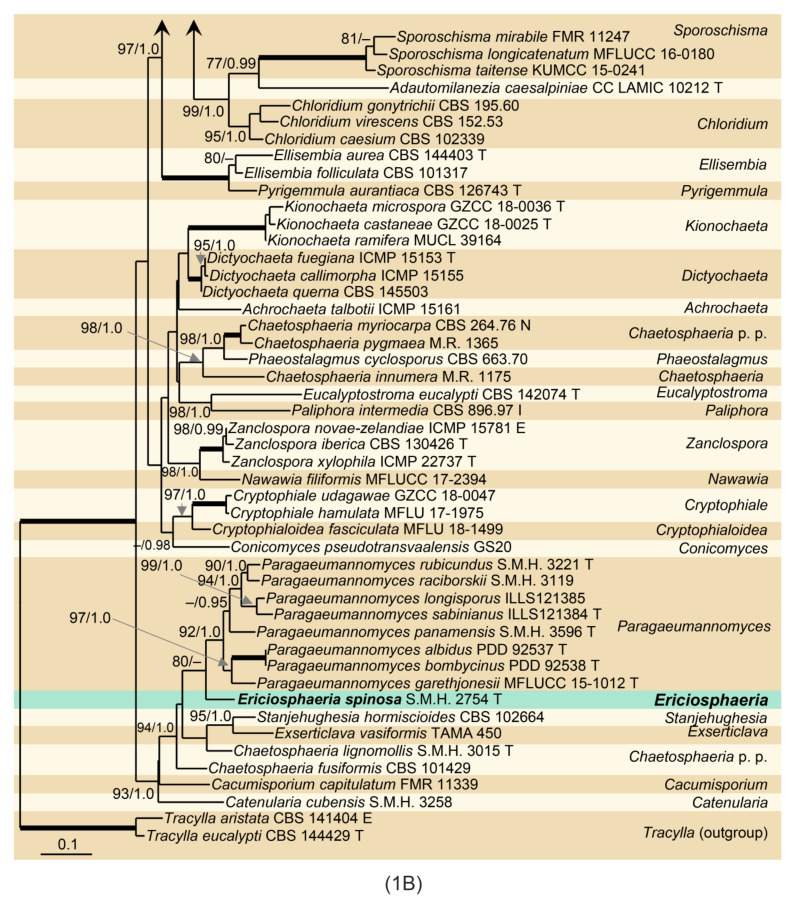
(**A**) Phylogenetic tree based on the combined ITS-28S rDNA sequences constructed by maximum likelihood (RAxML) of selected members of the Chaetosphaeriaceae. Species names given in bold and placed in green boxes are taxonomic novelties. T, E, I, N and P indicate ex-type, ex-epitype, ex-isotype, ex-neotype and ex-paratype strains; asterisk (*) indicates ex-type of *Tainosphaeria obclavata* (=*Phialogeniculata guadalcanalensis*). Thickened branches indicate branch support with ML BS = 100% and PP values = 1.0. Branch support of nodes ≥ 75% ML and ≥0.95 PP is indicated above or below branches. (**B**) Phylogenetic tree based on the combined ITS-28S rDNA sequences of the Chaetosphaeriaceae (continued). For legend refer to (**A**). Abbreviation: p.p. after a genus name (pro parte).

Phylogenetic analysis was based on the combined ITS-28S sequences of 109 members of the Chaetosphaeriaceae. The phylogenetic trees generated by BI and ML analyses were largely congruent; they only slightly varied in the topology of statistically unsupported clades. In the ML and BI analyses, nodes with support values of ≥75% ML BS and ≥0.95 BI PP were considered well-supported. The ML tree is shown in Figure 1. The Chaetosphaeriaceae included 50 lineages representing genera or natural groups of species. *Menisporopsis* was resolved as two separate, well-supported clades. The lineage containing *M. theobromae* and the other four *Menisporopsis* species represents the core of the genus (97/1.0). Two strains of *Menisporopsis novae-zelandiae* from Venezuela (CBS 109474, CBS 1094746), two morphologically similar isolates from Thailand (CBS 147509, CBS 147510) and strains from Japan (CBS 694.74) and Nepal (MUCL 43189) clustered as another lineage (100/1.0). They are proposed as the new genus *Arcuatospora*. The *Tainosphaeria* complex includes five lineages that correspond to particular morphologies, namely *Anacacumisporium*, *Phialogeniculata, Tainosphaeria* s. str., and two unnamed clades described as new genera, *Phialoturbella* and *Flectospora*, below. The *Tainosphaeria* s. str. subclade (100/1.0) includes *T. crassiparies*, *T. jonesii*, *T. monophialidica*, *T. siamensis* and a new species, *T. cecropiae* (CBS 101687). Two former *Tainosphaeria* species, *T. aseptata* and *T. lunata*, and the isolate ICMP 23826 formed a distinct subclade (90/1.0) that is introduced as *Phialoturbella*. *Tainosphaeria obclavata* (=*Phialogeniculata guadalcanalensis*) was resolved as a basal lineage to *Phialoturbella*. A chloridium-like dematiaceous hyphomycete represented by the strain CBS 112964 and another strain ICMP 23840 nested in the *Tainosphaeria* clade as monophyletic lineage (95/1.0), are introduced here as the new genus *Flectospora*. The genus *Chloridium* s. str. was resolved as a separate, well-supported clade (95/1.0). Two *Codinaea* species, *C. dimorpha* (CBS 140002, NBRC 33230) and *C. triseptata* (CBS 487.92, IMI 353690) grouped with *M. sympodialis* (MFLUCC 18-0153) in a well-supported *Multiguttulispora* clade (100/1.0). The type strain of *Chaetosphaeria spinosa* (S.M.H. 2754) clustered as a sister to *Paragaeumannomyces* (80/-) in the well-supported *Paragaeumannomyces* clade (93/1.0) together with *Exserticlava vasiformis* (TAMA 450), *Stanjehughesia hormiscioides* (CBS 102664), *Catenularia cubensis* (S.M.H. 3258) and two *Chaetosphaeria* species.

### 3.2. Taxonomy

*Arcuatospora* Réblová & Hern.-Restr., gen. nov. MycoBank MB 839474.

Etymology: *Arcuatus* (L) bent, curved into the shape of a bow, *spora* (L) spore, referring to falcate conidia.

Type species: *Arcuatospora novae-zelandiae* (S. Hughes & W.B. Kendr.) Réblová & Hern.-Restr.

Description: Colonies on the natural substrate effuse, hairy, mycelium superficial, composed of setose synnemata. Anamorph: Setae erect, straight, arise singly from a discoid, pseudoparenchymatous subiculum, dark brown to black, opaque, thick-walled, paler and thinner-walled at the apex, apex sterile, broadly rounded, occasionally terminating into a phialide. Conidiophores macronematous, synnematous, closely bound, parallel, unbranched, brown, synnemata arise around the base of the seta, surrounds the seta, diverge from it towards their apices and become unilateral. Conidiogenous cells integrated, terminal, mono- or polyphialidic, extending percurrently and sympodially, paler than the conidiophores; collarettes subhyaline, cup-shaped or funnel-shaped. Conidia falcate, slightly truncate at the base with a basal scar, 1-septate, hyaline, with a straight or gently curved setula at each end, inserted terminally at the apex, subterminally at the base, conidia accumulate in slimy fascicles. Teleomorph: not observed.

Habitat and geographical distribution: Members of the genus are saprobes on decaying leaves, petioles and fruits of various host plants. They have been reported worldwide from freshwater and terrestrial biotopes in subtropical and tropical geographical areas [6,9,63,64,65,66,67,68,69].

Note: Phylogenetic analysis of ITS-28S data set indicates that *Menisporopsis* is a polyphyletic genus (Figure 1). *Menisporopsis novae-zelandiae* and other four isolates are grouped as a strongly supported clade unrelated to the lineage containing *M. theobromae* and other species of *Menisporopsis*. The transversely 1-septate conidia with simple setulae and synnemata surrounding the seta becoming unilateral towards their apices are the main diagnostic characteristics of this newly discovered group. Based on the phylogenetic evidence supported by morphological data, *M. novae-zelandiae* and four other isolates are segregated into a new genus *Arcuatospora*.

**Figure 2:**
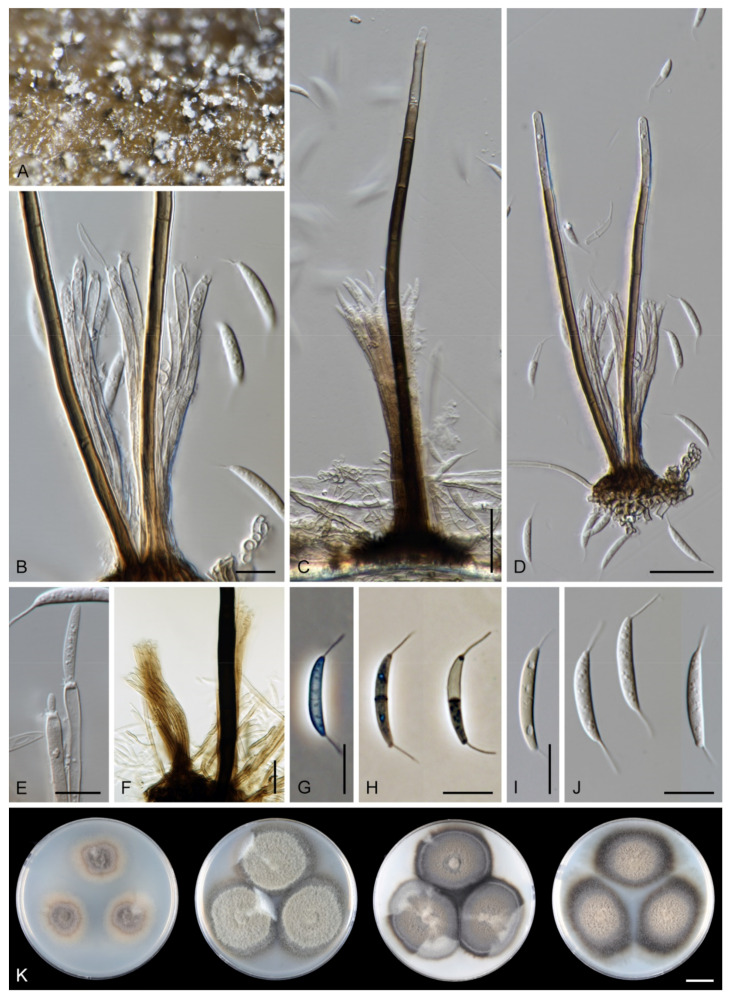
*Arcuatospora novae-zelandiae*. (**A**) colony on CMA with *Urtica* stems (**B**) synnemata around the setae, in detail (**C, D**) Setae with unilateral synnemata (**E**) conidiogenous cells (**F**) synnemata growing with and without setae (**G**–**J**) conidia (**K**) colonies on CMD, MLA, OA and PCA after 4 weeks (from left to right). Images: (**A**–**E**,**J**) CBS 109476; (**F**–**I**,**K**) CBS 109475; (**A**–**K**) on CMA with *Urtica* stems after 4 weeks. Scale bars: (**B**,**E**,**G**–**J**) 10 μm; (**C**,**D**,**F**) 20 μm; (**K**) 1 cm.

*Arcuatospora novae-zelandiae* (S. Hughes & W.B. Kendr.) Réblová & Hern.-Restr., comb. nov. MycoBank MB 839475. (Figure 2).

Basionym: *Menisporopsis novae-zelandiae* S. Hughes & W.B. Kendr., N. Z. J. Bot. 6: 369. 1968.

Characteristics in culture: On CMD colonies 24–28 mm diameter, circular, flat, margin entire, velvety-lanose, zonate, beige-grey, brown towards the margin with a beige outer zone, reverse dark brown. On MLA colonies 55–58 mm diameter, circular, raised, margin entire, lanose, floccose, funiculose, finely furrowed, olivaceous brown, dark olivaceous grey at the margin, diffusing dark amber pigment in the agar, reverse dark olivaceous brown. On OA colonies 60–62 mm diameter, circular, flat, margin entire, lanose, floccose centrally, mucoid to cobwebby towards the margin, dark olivaceous grey with irregular grey spots, reverse of the same colour. On PCA colonies 63–64 mm diameter, circular, slightly convex with a flat margin, margin entire to weakly fimbriate, lanose, floccose, funiculose centrally, mucoid to cobwebby towards the periphery, olivaceous brown, dark olivaceous grey at the margin, diffusing pale ochre pigment in the agar from the colony margin, reverse dark olivaceous brown. Sporulation was moderate on OA, absent on CMD, MLA and PCA.

Colonies on CMA with *U. dioica* stems effuse, hairy, mycelium composed of branched, septate, brown hyphae 1.5–3.5 μm diam. Anamorph: Setae 243–400 × 7.5–10.5 μm, 4–6.5 μm wide above the base, cylindrical or slightly widening from the base towards the middle, then gradually tapering towards the apex, solitary, erect, straight, dark brown to black, opaque, thick-walled, pale brown to subhyaline and thinner-walled at the apex, apex sterile, broadly rounded. Conidiophores 62–136 × 1.5–2.5 μm, macronematous, synnematous, closely bound, parallel, unbranched, erect, straight, arise in a group of 6–20 in separate bundles or surround the seta, diverge from it towards their apices and become unilateral, apically slightly bent when freed from the seta, pale brown, subhyaline at the apex, synnemata 7.5–14 μm wide near the base. Conidiogenous cells 12.5–25.5 × 3–3.5 μm, tapering to ca. 2(–2.5) μm below the collarette, integrated, terminal, monophialidic, occasionally extending percurrently, cylindrical-ovoid, pale brown, subhyaline towards the apex; collarettes 2.5–3.5 μm wide, ca. 1.5–2 μm deep, subhyaline, cup-shaped to funnel-shaped. Conidia 15.5–19.5 × 2–3 μm (mean ± SD = 17.8 ± 1.0 × 2.5 ± 0.4 μm), falcate, slightly truncate at the base with a basal scar, 1-septate, hyaline, with a straight or gently curved setula at each end 4.5–7.5 μm long, inserted terminally at the apex, subterminally at the base, conidia accumulate in slimy colourless fascicles. Teleomorph: not observed.

Specimens examined: VENEZUELA, Estado Miranda, in tropical cloud forest ‘Colonia Tovar’, on fallen decaying leaf of *Nectandra* sp., 25 November 2000, R.F. Castañeda-Ruíz & T. Itturiaga USB C00/77-3 (culture CBS 109474); Ibid., (culture CBS 109476).

Habitat and geographical distribution: Saprobe on decaying leaves and petioles, occasionally on fruits of a variety of hosts including *Beilschmiedia tarairi*, *Cinnamomum osmophloeum*, *C. zeylanicum*, *Cryptocarya mackinnoniana*, *Knightia excelsa*, *Nectandra* sp., *Ocotea leucoxylon*, *O. nemodaphne*, *Passania kawakamii*, *Quercus germana*, *Q. xalapensis*, *Quercus* sp. and palms. It has been reported in Australia, Brazil, Costa Rica, Cuba, Ecuador, Mexico, New Zealand, Taiwan and Venezuela [6,9,63,64,65,66,67,68,69,70].

Note: For a description on the natural substrate, see Hughes and Kendrick [9]. The two specimens examined in this study originate from Venezuela [6]. The conidial size of *A. novae-zelandiae* recorded by several authors differs and reflects either significant intraspecific variability or interspecific differences. Our observations of conidia in culture (15.5–19.5 × 2–3 μm with setulae 4.5–7.5 μm long) correspond to the conidia from nature in the protologue (15–18 × 2.4–3.1 μm with setulae 4.3–5.7 μm long) [9]. Cruz et al. [70] reported specimens of *A. novae-zelandiae* on decaying leaves and petioles in Brazil with smaller conidia and shorter setulae (9.5–11 × 1–1.5 μm, setulae 3–5 μm long). Two of our Thailand strains (CBS 147509, CBS 147510), tentatively identified as *A. novae-zelandiae*, have longer conidia (18.5–23 × 2.5–3.5 μm) with longer setulae (6–10 μm) and correspond to the measurements given for this species by Matsushima [71].

DNA sequence data of six strains initially identified as *A. novae-zelandiae* suggest it is a species complex with three cryptic species, i.e., *A. seorsa*, introduced for the long-spored variant, and two *Arcuatospora* spp. CBS 694.74 and MUCL 43189 treated as separate species. *Arcuatospora novae-zelandiae* with shorter conidia from Brazil [70], for which DNA data are not available, probably represents another undescribed species based on morphology. Based on many published records, *A. novae-zelandiae* appears to be a common species with a widespread geographical distribution in subtropical and tropical regions. Because of the discovered genetic variability of the *A. novae-zelandiae* species complex, these records need to be verified using molecular data.

*Arcuatospora novae-zelandiae* is closely related to *A. seorsa*; for their comparison, see notes to the latter species.

**Figure 3:**
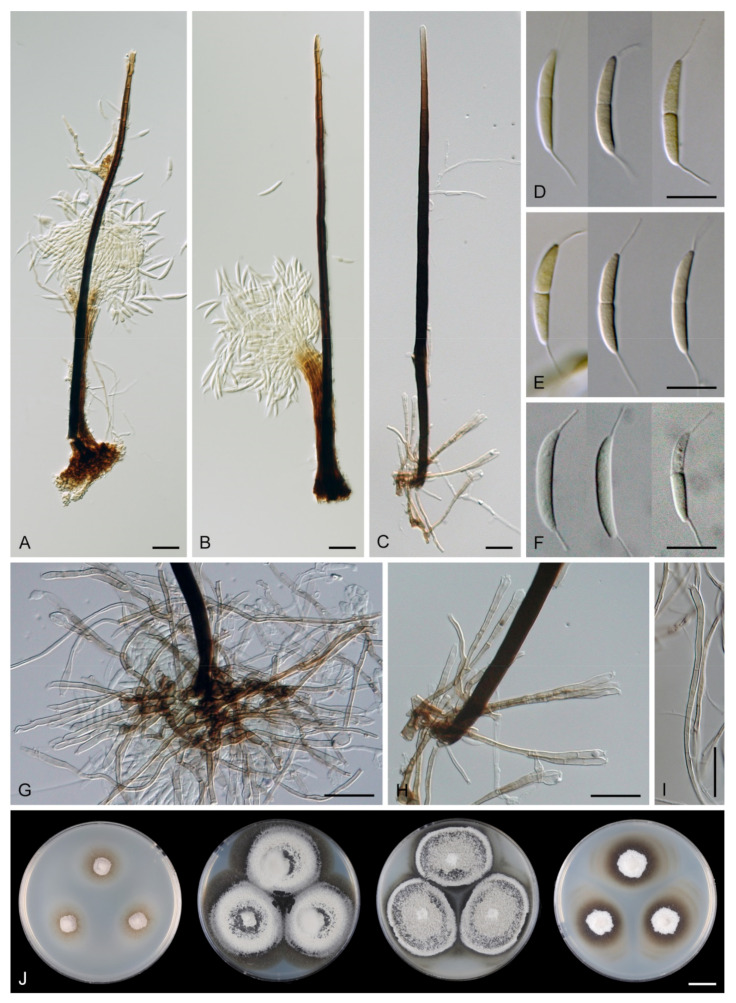
*Arcuatospora seorsa*. (**A**–**C**) setae with synnemata (**D**–**F**) conidia (**G**,**H**) base of the seta with conidiophores (**I**) conidiophore **(J**) colonies on CMD, MLA, OA and PCA after 4 weeks (from left to right). Images: (**A**,**B**,**D**,**E**,**J**) CBS 147510 ex-type; (**C**,**F**–**I**) CBS 147509; (**A**,**B**,**D**,**E**) from nature; (**C**,**F**–**I**) on PCA after 4 weeks. Scale bars: (**A**–**C**,**G**–**I**) 20 μm; (**D**–**F**) 10 μm; (**J**) 1 cm.

*Arcuatospora seorsa* Réblová & Hern.-Restr., sp. nov. MycoBank MB 839476. (Figure 3).

Etymology: *Seorsa* (L), apart from, segregated from a similar species *A. novae-zelandiae*.

Type: THAILAND, Nakhon Nayok province, Khao Yai National park, Princess trail, alt. 720 m, N 14°28′ E 101°22′, on fallen decaying leaf, 19 August 2001, M. Réblová & N. Hywel-Jones M.R. 2364/TH 378 (holotype PRA-19894, ex-type strain CBS 147510).

Description on the natural substrate: Colonies effuse, hairy, dark brown, composed of setose synnemata. Anamorph: Setae 320–457 × 7–9(–10) μm, 4–5 μm wide above the base, widening from the base towards the middle, then gradually tapering towards the apex, arise singly from a reddish-brown, discoid pseudoparenchymatous subiculum 35–40 μm diameter, erect, straight, dark brown, opaque, thick-walled, pale brown and thinner-walled at the apex, apex sterile, bluntly rounded. Conidiophores 122–158 × 2–3(–3.5) μm, macronematous, synnematous, closely bound, parallel, unbranched, erect, straight, arise in a group of 10–30 from the subiculum around the base of the seta, surround the seta, diverge from it towards their apices and become unilateral, apically slightly bent when freed from the seta, brown to reddish-brown, paler towards the apex, synnemata 11–22(–27) μm wide near the base. Conidiogenous cells 12.5–30(–34) × 3–4.5 μm, tapering to ca. 1.5 μm below the collarette, integrated, terminal, mono- occasionally polyphialidic with 1–2(–4) lateral openings, extending sympodially, cylindrical-ovoid, pale brown to subhyaline, old phialides bearing persistent remnants of the collarettes; collarettes 2–2.5 μm wide, ca. 1.5 μm deep, subhyaline, funnel-shaped. Conidia 18.5–23 × 2.5–3.5 μm (mean ± SD = 20.4 ± 1.1 × 3.1 ± 0.2 μm), falcate, slightly truncate at the base with a basal scar, 1-septate, sometimes slightly constricted at the septum, hyaline, with a straight or gently curved setula at each end 6–10 μm long, inserted terminally at the apex, subterminally at the base, conidia accumulate in slimy colourless to pale straw fascicles. Teleomorph: not observed.

Characteristics in culture: On CMD colonies 14–16 mm diameter, circular, convex, flat margin, margin entire, lanose centrally, mucoid towards the periphery, white-beige, pale brown towards the margin, with a white-isabelline outer zone of submerged growth, reverse dark brown. On MLA colonies 80–82 mm diameter, circular, convex, flat margin, margin entire, velvety-lanose, floccose, appearing powdery, locally mucoid, cobwebby to mucoid at the margin, furrowed, white to white-grey, dark olivaceous brown at the margin or when mucoid, reverse dark olivaceous brown. On OA colonies 75–76 mm diameter, circular, raised, margin entire, velvety-lanose, floccose, funiculose, with an intermediate mucoid zone, beige-grey, dark olivaceous grey at the margin and when mucoid, reverse dark olivaceous grey. On PCA colonies 42–45 mm diameter, circular, convex, flat margin, margin entire, lanose, floccose, mucoid towards the periphery, white centrally, brown to olivaceous brown at the margin, reverse dark brown. Sporulation was moderate on PCA, absent on CMD, MLA and OA.

Colonies on PCA effuse, hairy, mycelium composed of branched, septate, subhyaline to brown hyphae, becoming encrusted upon aging, 1.5–3 μm diam. Anamorph: Setae, conidiophores, conidiogenous cells and conidia similar to those from nature. Setae 340–390 × 8–10 μm, 4.5–5 μm wide above the base. Conidiophores 50–82 × 2–2.5(–3) μm, synnematous, sometimes branched near the base, solitary or arise in a group of 2–6 in separate bundles or along the seta, pale brown. Conidiogenous cells 12–17 × 2.5–4 μm, tapering to ca. 1.5–2 μm below the collarette, mono- or polyphialidic with 1(–2) lateral openings; collarettes 2.5–3.5 μm wide, ca. 2 μm deep. Conidia 16.5–19 × 2.5–3 μm (mean ± SD = 17.9 ± 0.8 × 2.6 ± 0.2 μm), 0–1-septate, with straight or gently curved setula at each end 4–6 μm long, conidia accumulate in slimy colourless fascicles. Teleomorph: not observed.

Other specimen examined: THAILAND, Nakhon Nayok province, Khao Yai National park, Mor Singh To trail, N 14°26′ E 101°22′, on decaying fruit of *Dipterocarpus* sp., 16 August 2001, M. Réblová & N. Hywel-Jones M.R. 2486/TH 116 (strain CBS 147509).

Habitat and geographical distribution: Saprobe on decaying fruits and leaves of *Cinnamomum japonicum*, *Dipterocarpus* sp., *Lithocarpus edulis*, *Litsea japonica*, known in Thailand and Japan (as *A. novae-zelandiae*) ([71], this study).

Note: *Arcuatospora novae-zelandiae* closely resembles *A. seorsa* but differs in longer setae (220–820 μm) and shorter conidia (15–18 μm) with shorter setulae (4.3–5.7 μm). Matsushima [71] reported three collections of *A. novae-zelandiae* with conidia (16.5–)18–20 × 2.2–3 μm and setulae 5–8 μm long on decaying leaves of evergreen trees in Japan. Although Japanese collections and cultures are not available for study, based on their original description they probably represent *A. seorsa*. They are well-comparable with *A. seorsa* except for the setae that are up to 600 μm long.

**Figure 4:**
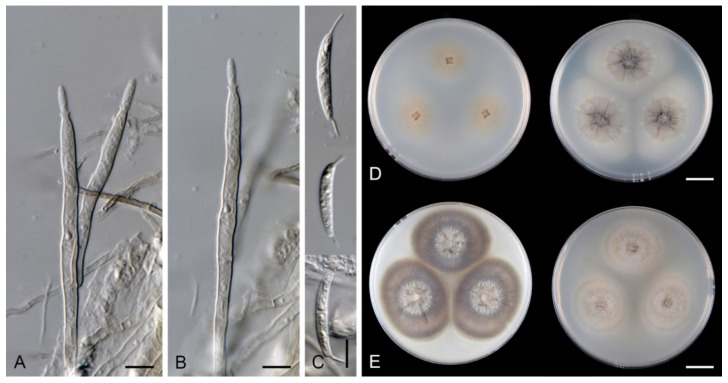
*Arcuatospora* sp 1. (CBS 694.74). (**A**,**B**) conidiophores (**C**) conidia (**D**,**E**) colonies. Images: (**A**–**C**) on CMA with *Urtica* stems after 7 weeks; (**D**) colonies on CMD and MLA after 4 weeks (from left to right); (**E**) colonies on OA and PCA after 4 weeks (from left to right). Scale bars: (**A**–**C**) 5 μm; (**D**,**E**) 1 cm.

*Arcuatospora* sp. 1 CBS 694.74 (Figure 4).

Characteristics in culture: On CMD colonies 21–23 mm diameter, circular, flat, margin entire, aerial mycelium scarce, restricted to the inoculation block, funiculose, colony mucoid, pale brown, reverse of the same colour. On MLA colonies 64–65 mm diameter, circular, flat, margin entire, sparsely lanose, funiculose centrally, mucoid towards the periphery, deeply furrowed, pale grey-brown, reverse of the same colour. On OA colonies 48–50 mm diameter, circular, somewhat crateriform, flat margin, margin entire, funiculose centrally, mucoid towards the periphery, furrowed, zonate, beige-grey at the centre, dark brown towards the margin, reverse olivaceous brown with an ochre tinge. On PCA colonies 62–64 mm diameter, circular, raised, margin entire, lanose, funiculose, furrowed, zonate, white-beige becoming olivaceous beige, pale pink-ochre pigment diffusing in the agar, reverse of the same colour. Sporulation was absent on all media.

Colonies on CMA with *U. dioica* stems effuse, mycelium composed of hyaline to brown, smooth hyphae 1–2 μm diam. Setae not observed. Conidiophores simple or branched, reduced to single conidiogenous cells, pale brown, smooth. Conidiogenous cells 25–42 × 3–3.5 μm, tapering to 1–1.5 μm below the collarette, monophialidic, cylindrical to lageniform, pale brown; collarettes 2–3 μm wide, 0.5–1 μm deep, funnel-shaped. Conidia 15–20 × 3–3.5 μm (mean ± SD = 16.9 ± 1.8 × 3.0 ± 0.2 μm), falcate, truncate at the basal hilum, 0–1-septate, hyaline, with straight or curved setula at each end 1–5.5 μm long, inserted terminally at the apex, subterminally at the base. Teleomorph: not observed.

Specimen examined: JAPAN, Kagoshima Prefecture, Ōsumi Islands, Tanagashima Island, Mt. Amagi, decaying leaf of an angiosperm tree, 23 November 1968, T. Yokoyama (culture CBS 694.74).

Habitat and geographical distribution: Saprobe on leaf litter, known only in Japan.

Note: The strain CBS 694.74 sporulated weakly only on *Urtica* stems on CMA. Due to the lack of morphological characteristics such as synnemata and setae, this strain is accepted as *Arcuatospora* sp. 1 and distinguished from other species by DNA data. In the phylogenetic tree, it grouped as a sister to the *Arcuatospora* sp. 2 MUCL 43189.

**Figure 5:**
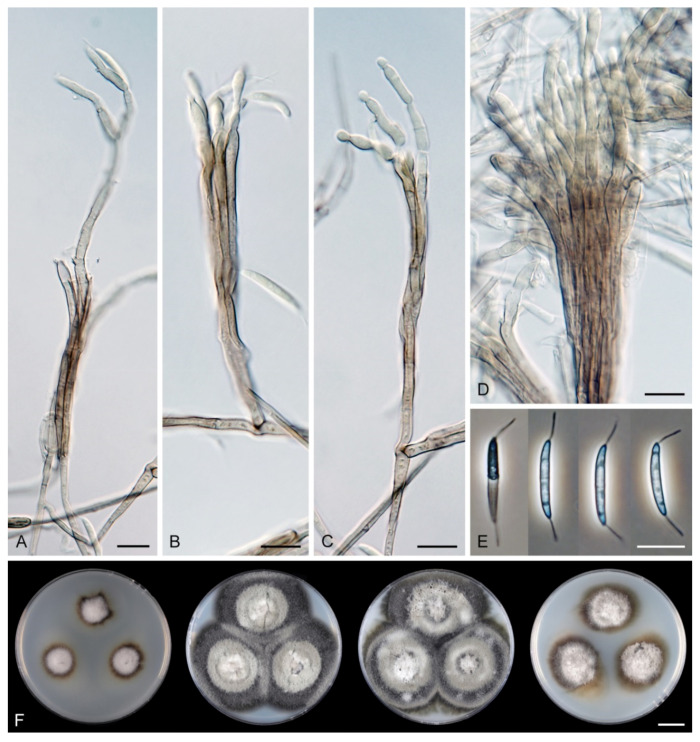
*Arcuatospora* sp 2. (MUCL 43189). (**A**–**D**) synnemata (**E**) conidia (**F**) colonies on CMD, MLA, OA and PCA after 4 weeks (from left to right). Images: (**A**–**E**) on PCA after 8 weeks. Scale bars: (**A**–**E**) 10 μm; (**F**) 1 cm.

*Arcuatospora* sp. 2 MUCL 43189 (Figure 5).

Characteristics in culture: On CMD colonies 55–60 mm diameter, circular, convex, margin entire, lanose, mucoid towards the margin, white, brown at the margin, reverse dark brown. On MLA colonies 50–56 mm diameter, circular, raised, margin entire, lanose, somewhat funiculose at the centre, furrowed, white to mouse grey, dark grey to nearly black towards the periphery, reverse dark grey to black. On OA colonies 60–62 mm diameter, circular, flat to slightly raised, lanose, floccose, funiculose, with zones of sparse growth, white to olivaceous grey, dark olivaceous grey towards the periphery, reverse dark olivaceous grey. On PCA colonies 68–70 mm diameter, circular, convex, margin entire, lanose, funiculose centrally, white-grey, dark brown towards the margin, reverse dark brown. Sporulation was sparse on PCA after prolonged incubation (>8 weeks), absent on CMD, MLA, and OA.

Colonies on PCA effuse, hairy, mycelium composed of branched, septate, subhyaline to pale brown hyphae 1.5–3 μm diam. Anamorph: Setae absent. Conidiophores 64–102 × 2–2.5 μm, macronematous, synnematous, sometimes branched near the base, closely bound, parallel, looser towards their apices, erect, straight or slightly flexuous, arise in a group of 4–15 in separate bundles, pale brown, subhyaline at the apex, synnemata 6.5–12 μm wide near the base. Conidiogenous cells 11.5–18.5(–25.5) × 3–3.5 μm, tapering to 1.5–2 μm below the collarette, integrated, terminal, mono- occasionally polyphialidic with 1(–2) lateral openings, extending percurrently and sympodially, cylindrical-ovoid, pale brown, subhyaline towards the apex; collarettes ca. 2.5 μm wide, ca. 1.5 μm deep, subhyaline, cup-shaped or funnel-shaped. Conidia (13.5–)15–17.5 × 2–2.5 μm (mean ± SD = 16 ± 0.9 × 2.2 ± 0.2 μm), falcate, slightly truncate at the base with a basal scar, 1-septate, hyaline, with straight or gently curved setula at each end 5–6 μm long, inserted terminally at the apex, subterminally at the base, conidia accumulate in slimy colourless fascicles. Teleomorph: not observed.

Specimen examined: NEPAL, 2001, L. Olivier (culture MUCL 43189).

Habitat and geographical distribution: The substrate and host are unknown; the strain originates from Asia, Nepal.

Note: Although the strain MUCL 43189 shares with *A. novae-zelandiae* morphological characteristics of conidiophores, phialides and conidia, the phylogenetic analysis did not support their close relationship. When grown in culture, MUCL 43189 did not form setae, not even after prolonged incubation. In the conidial size, this strain corresponds to *A. novae-zelandiae*, although the conidia were slightly shorter and narrower in their lower range. In the absence of morphological traits such as setae, the isolate MUCL 43189 is referred to as *Arcuatospora* sp. 2 in this study. More material is needed to study this species under natural conditions.

*Ericiosphaeria* Réblová & Hern.-Restr., gen. nov. MycoBank MB 839477.

Etymology: *Ericius* (L) hedgehog, *sphaeria* (L), hedgehog-like, referring to ascomata covered with short, acute spines.

Type species: *Ericiosphaeria spinosa* (F.A. Fernández & Huhndorf) Réblová & Hern.-Restr.

Description: Colonies on the natural substrate composed of ascomata. Anamorph: Setae absent. Conidiophores semi-macronematous, simple, solitary. Conidiogenous cells integrated, terminal, monophialidic, ampulliform, hyaline; collarettes funnel-shaped. Conidia ellipsoidal to globose, aseptate, hyaline, accumulating in slimy fascicles (known only in culture; adapted from Fernández and Huhndorf [3]). Teleomorph: Ascomata perithecial, non-stromatic, superficial, solitary or in small groups, globose to ovoid, papillate, dark brown to nearly black, setose. Setae rigid, dark brown, opaque, simple, acute, aseptate, never conidiogenous. Ostiolar canal periphysate. Ascomatal wall fragile, carbonaceous, two-layered. Paraphyses persistent, branching, hyaline, septate, longer than asci. Asci unitunicate, cylindrical-clavate, with a non-amyloid apical annulus, eight-spored. Ascospores cylindrical to filiform, straight, sometimes bent to sigmoid, asymmetrical, rounded at the apical end, tapering towards the basal end, aseptate (probably transversely septate), hyaline, without mucilaginous sheath or appendages, arranged 3–4-seriately or in a fascicle within the asci.

Habitat and geographical distribution: Saprobes on decaying wood, known in the USA [3].

Note: The genus *Ericiosphaeria* is introduced for fungi with minute, dark ascomata covered by short, opaque, acute setae, two-layered ascomatal wall, scolecosporous, hyaline ascospores and anamorphs with phialidic conidiogenesis. The new genus is typified with *E. spinosa*, previously classified in *Chaetosphaeria* [3].

*Ericiosphaeria* is remarkably similar to *Paragaeumannomyces* [7,20] in the characteristics of asci, ascospores and setae, which are never conidiogenous, but differs in anatomy of the ascomatal wall. *Ericiosphaeria* has the wall two-layered, dark brown and carbonaceous compared to *Paragaeumannomyces* with three-layered ascomatal wall. The outer wall is usually coloured, ranging from white, yellow-white, ginger to reddish-brown or occasionally dark brown and is composed of globose to angular cells. The middle layer, on the other hand, is the typical ’chaetosphaeriaceous‘ ascomatal wall, which is melanized and composed of brown, brick-like cells. Both genera are comparable in the anamorphic characteristics, so far observed only in culture. *Paragaeumannomyces* has been linked with a craspedodidymum-like and chloridium-like synanamorphs [72], while *Ericiosphaeria* forms a chloridium-like anamorph [3]. Both genera were resolved as closely related taxa.

*Ericiosphaeria spinosa* (F.A. Fernández & Huhndorf) Réblová & Hern.-Restr., comb. nov. MycoBank MB 839488.

Basionym: *Chaetosphaeria spinosa* F.A. Fernández & Huhndorf, Fungal Divers. 18: 36. 2005.

Habitat and geographical distribution: Saprobe on decaying bark and wood of *Betula* sp., known only in North America in the USA (North Carolina, Texas) [3].

Note: For description, illustration and characteristics in culture, see Fernández and Huhndorf [3]. Although the ascospores were described as aseptate with numerous guttules, it is likely that the septa were obscured by the droplets as is often the case with similar ascospores in *Paragaeumannomyces* [20].

*Flectospora* Réblová & Hern.-Restr., gen. nov. MycoBank MB 839478.

Etymology: *Flectus* (L) bend, curved, *spora* (L) spore, referring to the curved conidia.

Type species: *Flectospora laminata* Réblová & Hern.-Restr.

Description: Colonies on the natural substrate effuse, hairy, mycelium semi-immersed, composed of conidiophores, occasionally ascomata. Anamorph: Setae absent. Conidiophores macronematous, mononematous, solitary, erect, straight or slightly flexuous, cylindrical, unbranched, thick-walled, septate, smooth, brown. Conidiogenous cells integrated, terminal, mono- or polyphialidic with several lateral apertures, extending percurrently and sympodially, paler than the conidiophores, conidia formed on single conidiogenous loci; collarettes funnel-shaped. Conidia ellipsoidal to obovoid, slightly curved, aseptate, hyaline, accumulate in slimy colourless fascicles. Teleomorph: Ascomata perithecial, non-stromatic, superficial, subglobose to conical, papillate or with a rostrate neck, dark brown, glabrous. Ostiole periphysate. Ascomatal wall two-layered, carbonaceous. Paraphyses persistent, septate, hyaline. Asci unitunicate, cylindrical-clavate, short-stipitate, ascal apex with a non-amyloid apical annulus, 8-spored. Ascospores ellipsoidal-fusiform, hyaline, transversely septate, smooth.

Habitat and geographical distribution: Saprobes on decaying wood, known so far in New Zealand and Thailand.

Note: *Flectospora* is established for ascomycetes with phialidic dematiaceous hyphomycete anamorphs with macronematous, simple conidiophores and hyaline, ellipsoidal to obovoid, aseptate, slightly curved conidia accumulating in slimy heads. The teleomorphs have glabrous perithecial ascomata, unitunicate asci, paraphyses, and hyaline, septate ascospores. The genus accommodates two species.

*Flectospora laminata* was initially considered the chloridium-like anamorph of *Chaetosphaeria hispida* [19], characterised by scolecosporous, septate, hyaline ascospores and ascomata covered with short, opaque spines. The holotype of *Ch. hispida* contains conidiophores of the chloridium-like fungus growing near the ascomata. Identical, sporulating conidiophores were obtained in culture (strain CBS 112964), from which the DNA sequences were derived. However, phylogenetic analysis of ITS-28S sequences, including the chloridium-like anamorph, *E. spinosa*, members of *Paragaeumannomyces* and other morphologically similar species, have raised suspicions that the chloridium-like fungus is not the anamorph but likely a contamination. The isolation into axenic culture occurred during the fieldwork in the Khao Yai forest in Thailand. Although the strain was derived from ascospores, the conidia were probably removed from the natural material by accident.

Although *Ch. hispida*, *E. spinosa* and *Paragaeumannomyces* are remarkably similar in characters to ascomata, asci and especially ascospores, they grouped in distantly related clades. The ex-type strain of *E. spinosa* S.M.H. 2754 and *Paragaeumannomyces* spp. clustered in one clade, while the ex-type strain of *Ch. hispida* CBS 112964 nested in the *Tainosphaeria* clade. It is difficult to reconcile such different teleomorphic features with the revealed phylogenetic relationships. Known teleomorphs of the *Tainosphaeria* clade have glabrous ascomata and symmetrical, ellipsoidal to fusiform, 0–several-septate ascospores ([3], this study). The results of the phylogenetic analysis prompted the revision of the holotype of *Ch. hispida* and has been found to be a species of *Paragaeumannomyces* [7,20]. Based on the phylogenetic arguments and critical evaluation of teleomorphic and anamorphic characteristics, we conclude that the chloridium-like presumed anamorph of *Ch. hispida* does not belong to its life cycle. Therefore, the hyphomycete growing in the holotype of *Ch. hispida* is transferred to the new genus *Flectospora* as *F. laminata* and a new combination for *Ch. hispida* in *Paragaeumannomyces* is proposed below.

**Figure 6:**
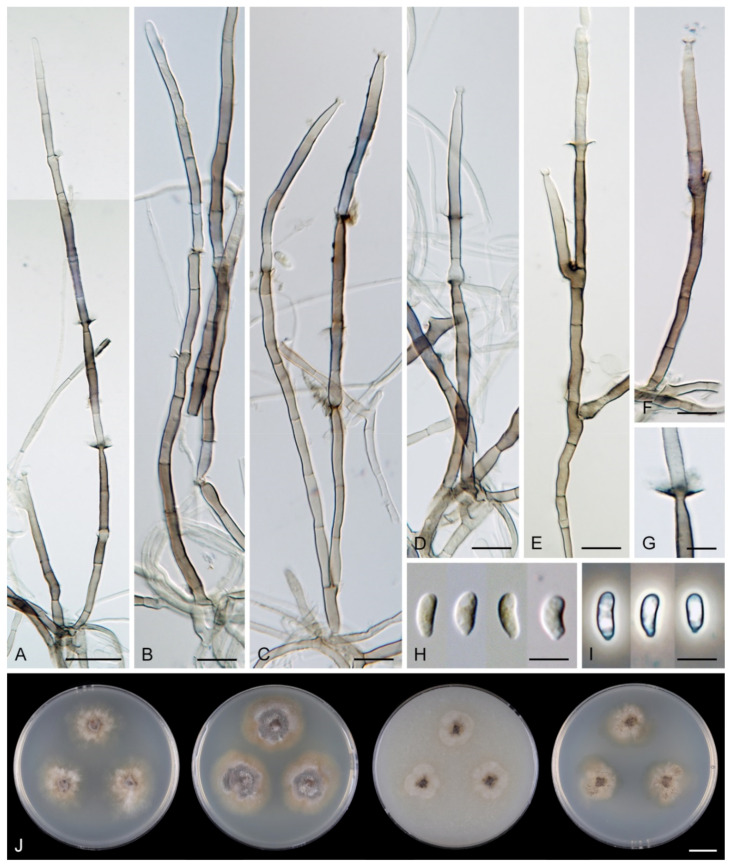
*Flectospora laminata*. (CBS 112964 ex-type). (**A**–**F**) conidiophores (**G**) saucer-shaped collarette (**H**,**I**) conidia (**J**) colonies on CMD, MLA, OA and PCA after 4 weeks (from left to right). Images: (**A**–**I**) on PCA after 4–6 weeks. Scale bars: (**A**) 20 μm; (**B**–**F**) 10 μm; (**G**–**I**) 5 μm; (**J**) 1 cm.

*Flectospora laminata* Réblová & Hern.-Restr., sp. nov. MycoBank MB 839479 (Figure 6).

Etymology: *Lamina* (L), plate, -*ata* (L) possessing, referring to the saucer-shaped collarettes that remain on the conidiophore after the phialide extends percurrently.

Type: THAILAND, Nakhon Nayok Province, Khao Yai National Park, Bung Phai trail ca. 5 km NW from Khao Yai forest Headquarters on a way to Pak Chong, alt. 750 m, 14°28′ N 10°23′ E, on decaying wood, 6 September 2001, M. Réblová, G. J. Samuels & R. Nasit M.R. 2220/TH 511 (holotype PRA-19895 as dried culture, voucher PRM 900543 holotype of *Chaetosphaeria hispida*, culture ex-type CBS 112964 = DAOM 231140).

Description on the natural substrate: Colonies effuse, hairy. Anamorph: Setae absent. Conidiophores 46–82 μm long, (3–)3.5–4 μm wide near the base, macronematous, mononematous, solitary, erect, straight or slightly flexuous, cylindrical, unbranched, thick-walled, brown, paler upwards, 4–6-septate, darker brown at the septa. Conidiogenous cells 15–35 × 3–4 μm, tapering to ca. 1.5 μm below the collarette, integrated, terminal, mono- or polyphialidic with 2–5 lateral apertures arising from the sympodial proliferation; collarettes 2.5–3 μm wide, 2–2.5(–3) μm deep, funnel-shaped, hyaline. Conidia (4.5–)5–6 × 2.5–3 μm (mean ± SD = 5.5 ± 0.3 × 2.6 ± 0.1 μm), ellipsoidal to obovoidal, slightly curved, truncate at the base, rounded apically, aseptate, hyaline, smooth. (adapted from Réblová and Seifert [19]). Teleomorph: not observed.

Characteristics in culture: On CMD colonies 20–22 mm diameter, circular, flat, margin rhizoidal, lanose, funiculose centrally, white-beige, with a pale ochre outer zone of submerged growth, reverse dark beige. On MLA colonies 22–27 mm diameter, circular, raised, margin entire, lanose, floccose, cobwebby towards the periphery, whitish to mouse grey with an ochre outer zone of submerged growth, pale ochre pigment diffusing into agar, reverse ochre. On OA colonies 12–13 mm diameter, circular, flat, margin entire to weakly undulate, cobwebby, white to ochre-beige with irregular dark grey spots due to aggregated sporulating conidiophores, with an olivaceous ochre outer zone of submerged growth, reverse pale ochre-beige. On PCA colonies 17–20 mm diameter, circular, circular, flat, margin entire, lanose to cobwebby, ochre-beige an olivaceous ochre outer zone of submerged growth, reverse of the same colours. Sporulation was abundant on MLA and PCA, sparse on CMD and OA.

Colonies on PCA effuse, hairy, mycelium composed of branched, septate, hyaline to pale brown hyphae 1–2 μm diam. Anamorph: Setae absent. Conidiophores 46–220 μm long, 2.5–3.5 μm wide above the base, macronematous, unbranched, occasionally branched in the lower part with 1–2 lateral branches, erect, straight or flexuous, septate, smooth, brown, pale brown to subhyaline towards the apex. Conidiogenous cells 13.5–33.5 × 3–4 μm, tapering to 1.5–2 μm below the collarette, integrated, terminal, mono- rarely polyphialidic with one lateral aperture, extending percurrently and sympodially, cylindrical, pale brown to subhyaline, paler towards the apex; collarettes at the tip of the conidiogenous cells 2.5–4 μm wide, 1.5–2.5 μm deep, funnel-shaped, subhyaline, intercalary collarettes that are remain on the conidiophore after the conidiogenous cell extend percurrently are saucer-shaped, usually dark brown and appear like widely open flaps surrounding the conidiophore, 8–8.5 μm wide, 1.5–2.5 μm deep. Conidia 5–6.5 × 2–3 μm (mean ± SD = 5.9 ± 0.4 × 2.4 ± 0.4 μm), ellipsoidal to obovoid, slightly curved, rounded at the apical end, tapering towards the base, base slightly truncate with a basal scar, hyaline, aseptate, smooth, conidia accumulate in slimy colourless fascicles. Teleomorph: not observed.

Habitat and geographical distribution: Saprobe on decaying wood, known only in Thailand.

Note: Although the conidiophores are unbranched on material from nature, they occasionally branch in the lower part when grown in the culture. The phialidic conidiogenous cells extend percurrently and form characteristic saucer-shaped collarettes that are persistent and appear like wide-open flaps, compared to the apical collarettes which are subhyaline and funnel-shaped. *Flectospora laminata* resembles two *Chloridium* species. *Chloridium curviellipticum* was described from a decaying petiole of palm in Peru and differs in longer conidia, 6.5–11 × 2–2.5(–3) μm [65]. *Chloridium reniforme* var. *minor* is known from Cuba on decaying wood and has shorter conidia, 3–4 × 1.2–1.8 μm [73].

**Figure 7:**
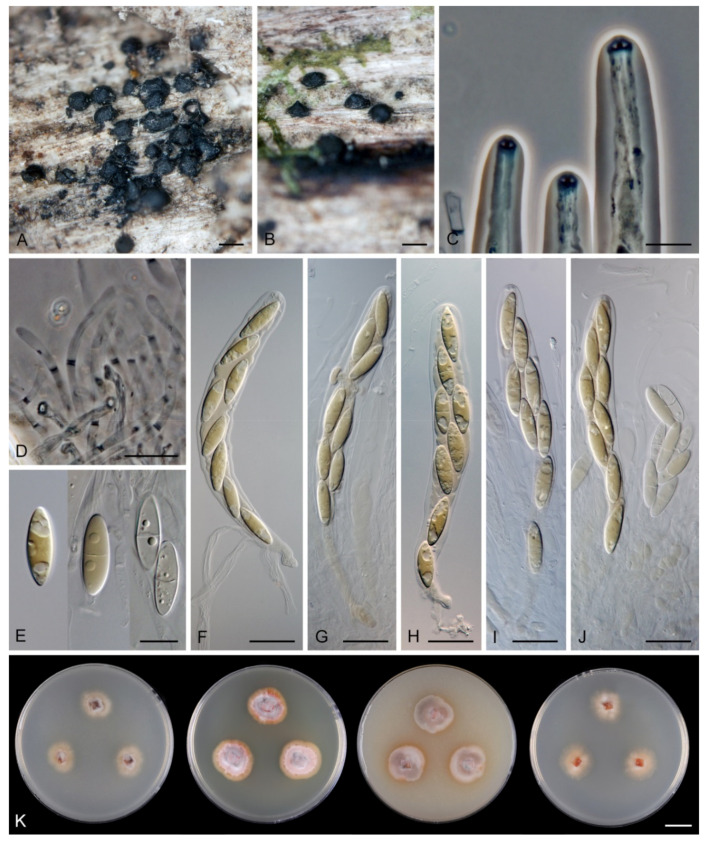
*Flectospora* sp. (ICMP 23840). (**A**,**B**) ascomata (**C**) ascal apices with apical rings (**D**) paraphyses (**E**) ascospores (**F**–**J**) asci (**K**) colonies on CMD, MLA, OA and PCA after 4 weeks (from left to right). Images: (**A**–**J**) from nature. Scale bars: (**A**,**B**) 250 μm; (**C**,**E**) 10 μm; (**D**,**F**–**J**) 20 μm; (**K**) 1 cm.

*Flectospora* sp. ICMP 23840 (Figure 7).

Description on the natural substrate: Anamorph: not observed. Teleomorph: Ascomata 250–310 μm diam, 290–350 μm high, non-stromatic, superficial to semi-immersed, subglobose to conical, papillate or with a rostrate neck, dark brown, glabrous, glossy. Ostiole periphysate. Ascomatal wall fragile, carbonaceous, 14–23 μm thick, two-layered. Outer layer consisting of dark brown, polyhedral cells with opaque walls. Inner layer consisting of several rows of thin-walled, hyaline cells. Paraphyses septate, hyaline, cylindrical, some cells slightly inflated, 2.5–5 μm wide, longer than the asci. Asci 130–150 × 12–15(–16) μm (mean ± SD = 142 ± 12.3 × 17.1 ± 0.6 µm), 106–130 μm (mean ± SD = 119.8 ± 10.4 µm) long in the sporiferous part, cylindrical-clavate, short-stipitate, apically obtuse, ascal apex with a non-amyloid apical annulus 4–5.5 μm wide, 2–2.5 μm high. Ascospores 19.5–25 × 5.5–6.5(–7) μm (mean ± SD = 21.2 ± 1.3 × 6.3 ± 0.4 µm), ellipsoidal-fusiform, hyaline, 0–1-septate, smooth, 2-seriate or obliquely uniseriate in the ascus.

Characteristics in culture: On CMD colonies 10–11 mm diameter, circular, convex, margin entire, lanose, whitish to light apricot, light ochre pigment diffusing into agar, reverse ochre-beige. On MLA colonies 14–16 mm diameter, circular, convex, margin entire, velvety-lanose, furrowed, white-grey to grey centrally, white-apricot towards the margin with a deep apricot outer zone of submerged growth, pale orange pigment diffusing into agar, reverse deep apricot, paler at the margin. On OA colonies 13–15 mm diameter, circular, convex, margin entire, lanose, furrowed, white-grey to pale apricot-grey, white towards the margin, pale apricot pigment diffusing into agar, reverse apricot. On PCA colonies 12–13 mm diameter, circular, flat, margin fimbriate, cobwebby, white-grey, pale apricot towards the margin, reverse pale ochre. Sporulation was absent on all media.

Specimen examined: NEW ZEALAND, West Coast region, Westland district, Haast, Jackson River valley, Lake Ellery track, on decaying wood, 11 March 2003, M. Réblová M.R. 2790/NZ 300A (ICMP 23840).

Habitat and geographical distribution: Saprobe on decaying wood, known only in New Zealand.

Note: This species is tentatively placed in *Flectospora*; in the phylogenetic analysis it grouped as a sister to *F. laminata* in a strongly supported clade. It is characterised by dark, glabrous, rostrate ascomata, ellipsoidal, hyaline, 0–1-septate ascospores and apricot pigment diffusing into the agar. Although anamorphs of the *Tainosphaeria* clade are conspicuous dematiaceous phialidic hyphomycetes such as *Anacacumisporium* [18], *Phialogeniculata* [16,17], *Phialoturbella* (this study) and *Tainosphaeria* [3], which readily sporulate in culture, the anamorph of the present species is unknown. On the natural substrate only ascomata occurred and the culture derived from ascospores remained sterile. The species is included in the study to further characterise the emerging *Tainosphaeria* clade.

*Multiguttulispora* C.G. Lin & J.K. Liu, Mycosphere 10: 681. 2019.

Type species: *Multiguttulispora dimorpha* (Toyaz. & Udagawa) Réblová & Hern.-Restr.

Emended description: Colonies on natural substrate effuse, hairy, mycelium partially superficial, partially immersed, composed of conidiophores. Anamorph: Setae absent. Conidiophores macronematous, mononematous, solitary or arise in groups of 2–3, straight or flexuous, unbranched, septate, smooth, dark brown, opaque and thick-walled, paler and thinner-walled towards the apex, with several lateral phialidic openings or fertile zones consisting of aggregated lateral openings along the conidiophore axis. Conidiogenous cells integrated, terminal, mono- and polyphialidic, extending sympodially over a short distance, cylindrical, paler than the conidiophore, often with persistent remnants of the collarettes; collarettes funnel-shaped, hyaline to subhyaline. Conidia ellipsoidal to oblong to ellipsoidal-fusiform, with an inconspicuous basal scar, transversely septate, hyaline, with a gently curved setula at each end, conidia accumulate in slimy fascicles. Synanamorph (formed only in culture): Setae absent. Conidiophores semi-macronematous, mononematous, pale brown, mostly reduced to single conidiogenous cells, which are monophialidic, integrated, terminal, pale brown, collarettes funnel-shaped. Conidia falcate-fusiform to navicular, with a basal hilum, aseptate, hyaline, without setulae, accumulate in colourless slimy masses. Teleomorph: not observed.

Habitat and geographical distribution: The holotype of *M. dimorpha* was isolated from the air in Japan, and other records confirm members of *Multiguttulispora* as saprobes on decaying plant material or endophytes. Species have a widespread geographical distribution in the tropical and temperate zones of Asia, Caribbean, Micronesia and North and South America [2,21,22,65,74,75,76].

Note: Lin et al. [2] introduced *Multiguttulispora*, typified with *M. sympodialis*, for dematiaceous hyphomycetes with macronematous conidiophores terminating in polyblastic, sympodial conidiogenous cells and septate, hyaline conidia with setulae. In the present phylogeny, the genus forms a monophyletic lineage containing two species, *M. dimorpha* and *M. triseptata*. Comparison of morphological characters and DNA sequences revealed *M. sympodialis* conspecific with *Codinaea dimorpha* [21] and its close relationship to *C. triseptata* [22]. Based on the phylogenetic evidence and morphological data, both *Codinaea* species are transferred to *Multiguttulispora,* and *M. sympodialis* is reduced to the synonymy of *M. dimorpha.*

**Figure 8:**
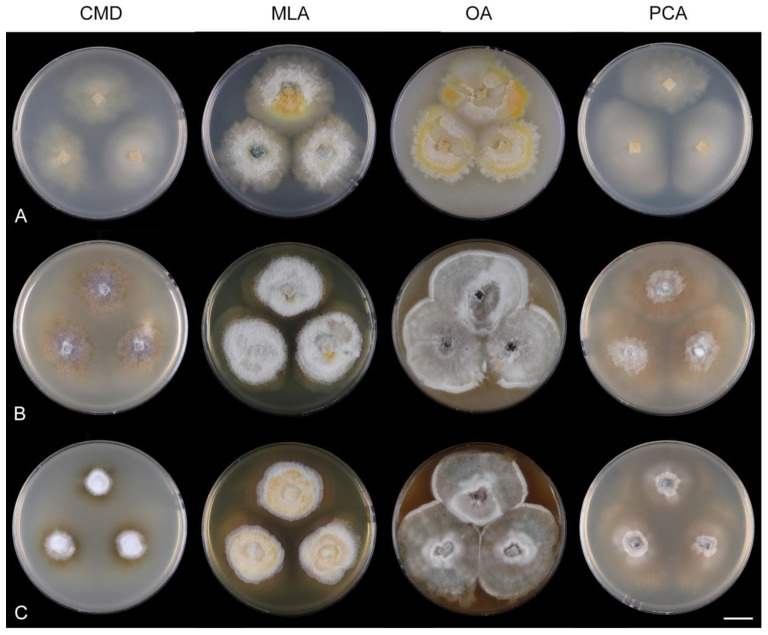
Colony morphology of *Multiguttulispora* after 4 weeks. (**A**) *M. dimorpha* CBS 140002 (**B**) *M. triseptata* IMI 353690 (**C**) *M. triseptata* CBS 487.92. Scale bar: (**A**–**C**) 1 cm.

*Multiguttulispora* is delimited to fungi with ellipsoidal to oblong to ellipsoidal-fusiform, hyaline, 3-septate conidia with a basal hilum and a gently curved setula at each end. The phialides extend sympodially over a very short distance; the phialidic apertures become densely aggregated at the tip of the conidiogenous cells and give the phialide a somewhat geniculate appearance. This feature is prominent in *M. dimorpha*. Solitary lateral openings or small fertile zones comprising several clustered phialidic openings are spread in irregular intervals along the conidiophore axis. The collarettes are easily detached and can remain attached to the bottom of the released conidia. Colonies of the analysed strains on the four growth media are compared in Figure 8.

**Figure 9:**
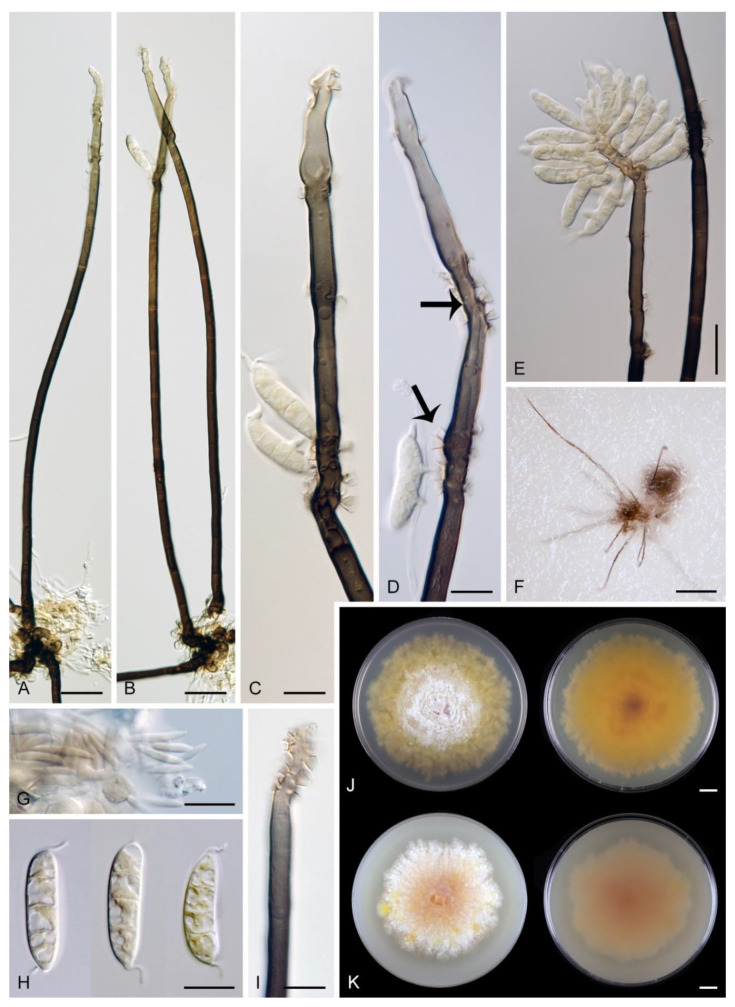
*Multiguttulispora dimorpha*. (CBS 140002). (**A**,**B**) conidiophores (**C**–**E**,**I**) upper fertile part of the conidiophores with lateral phialidic openings with collarettes (arrows indicate collarettes) (**F**) subsurface conidiophores arising in a group (**G**) microconidia (**H**) conidia (**J**,**K**) colonies (front and reverse). Images: (**A**–**E**,**G**–**I**) on SNA with pine needles after 8 weeks; (**F**) on PCA after 12 weeks; (**J**) on MLA after 8 weeks; (**K**) on OA after 8 weeks. Scale bars: (**A**,**B**) 25 μm; (**C**,**D**,**G**–**H**) 10 μm; (**F**) 250 μm; (**E**) 20 μm; (**J**,**K**) 1 cm.

*Multiguttulispora dimorpha* (Toyaz. & Udagawa) Réblová & Hern.-Restr., comb. nov. MycoBank MB 839480. (Figure 9).

Basionym: *Codinaea dimorpha* Toyaz. & Udagawa, Mycotaxon 13: 451. 1981.

Synonym: *Multiguttulispora sympodialis* C.G. Lin & J.K. Liu, Mycosphere 10: 681. 2019.

Characteristics in culture: On CMD colonies 25–30 mm diameter, circular, flat, margin fimbriate, cobwebby becoming mucoid, isabelline with a white tinge, with a prominent outer zone of submerged growth, reverse pale ochre-isabelline. On MLA colonies 45–47 mm diameter, circular, raised, margin fimbriate, velvety, floccose, wrinkled, appearing powdery, mucoid towards the margin, furrowed, white with irregular yellow-orange spots, with ochre to golden outer zone of submerged growth, pale yellow pigment diffusing into the agar, reverse yellow-orange with irregular cinnamon spots. On OA colonies 38–40 mm diameter, circular, flat, margin lobate, velvety, locally mucoid, zonate, deep orange-yellow with a pale peach tinge becoming peach-pink centrally, white towards the periphery, pale peach pigment diffusing into the agar, reverse peach. On PCA colonies 52–53 mm diameter, circular, flat, margin undulate, cobwebby becoming mucoid, pink-beige, reverse pale pink-beige. Sporulation was absent on CMD, MLA, sparse on PCA and OA after prolonged incubation.

Colonies on SNA with pine needles effuse, locally hairy, mycelium composed of branched, septate, hyaline hyphae 1.5–2.5 μm diam. Anamorph: Setae absent. Conidiophores 167–367(–550) μm long, 5–7(–7.5) μm wide near the base, macronematous, single or arise in groups of 2–4 from knots of hyphal cells, sometimes subsurface, erect, straight or flexuous, geniculate in the upper part, unbranched, septate, smooth, dark brown and thick-walled, paler and thinner-walled towards the apex. Fertile areas consisting of aggregated lateral openings are dispersed along the upper part of the conidiophore. Conidiogenous cells 21.5–89 × 5–6.5 μm, integrated, terminal, polyphialidic with numerous aggregated lateral openings while septa can be formed internally, extending sympodially over a short distance, cylindrical, medium to pale brown, subhyaline towards the apex, often with persistent remnants of the collarettes; collarettes 2.5–3.5 μm wide, 2–3.5 μm deep, funnel-shaped, subhyaline, often broken off in older culture and attached at the bottom of conidia. Conidia 22–27.5 × 6–7 μm (mean ± SD = 25.3 ± 1.6 × 6.4 ± 0.3 μm), ellipsoidal to oblong to ellipsoidal-fusiform, slightly curved, tapering towards both ends, with an inconspicuous basal scar, 3-septate, hyaline, with gently curved setula at each end, 4.5–6 μm long, positioned terminally at the apex and slightly subterminally at the base, conidia accumulate in slimy colourless fascicles. Synanamorph: Setae absent. Conidiophores 40–65 μm long, ca. 2.5 μm wide, usually arising around the base of conidiophores of the anamorph, semi-macronematous, pale brown, mostly reduced to single conidiogenous cells, 12.5–31 × 3–3.5 μm, tapering to 1.5 μm below the collarette, integrated, terminal, pale brown, cylindrical; collarettes 2.5–3 μm wide, 1.5–2 μm deep, funnel-shaped, pale brown. Conidia 9–13 × 1.5–2 μm (mean ± SD = 10.6 ± 1.4 × 1.8 ± 0.3 μm), falcate-fusiform to navicular, slightly obtuse at the apical end, truncate at the basal hilum, aseptate, hyaline, without setulae, accumulate in colourless slimy masses. Teleomorph: not observed.

Specimen examined: MALAYSIA, Sabah, on twigs of *Eucalyptus* sp., May 2014, M. J. Wingfield (culture CBS 140002).

Habitat and geographical distribution: Saprobe on decaying wood, leaves or fruits of *Inga* sp. and other unknown hosts, also isolated from the air. It is known in Japan, Malaysia, Peru and Thailand [2,21,65,76].

Note: Lin et al. [2] described this species as *M. sympodialis* from a decaying fruit in Thailand, strain MFLUCC 18-0153. Another conspecific strain CBS 140002 examined in this study is from a twig of *Eucalyptus* sp. in Malaysia (as *Dictyochaeta triseptata*) [76]. The latter strain corresponds to the protologue of *Codinaea dimorpha* [21] in all details except that the conidiophores were longer due to numerous sympodial extensions. *Codinaea dimorpha* was originally isolated from the air in Kobe in Japan [21]. The type strain (NHL 2891) is not available, but another strain of *C. dimorpha* NBRC 33230 (=IFO 33230 = KIH C 0300) also isolated from the air was deposited by N. Toyazaki, one of the co-authors of *C. dimorpha*. Although the culture of NBRC 33230 is currently unavailable, its and 28S rDNA sequences publicly accessible [30], were included in our study. Based on the results of the phylogenetic analysis and comparative morphology, *M. sympodialis* (MFLUCC 18-0153) and *C. dimorpha* (NBRC 33230, CBS 140002) are revealed as conspecific. Therefore, a new combination is proposed for *C. dimorpha* in *Multiguttulispora* and *M. sympodialis* is reduced to synonymy. Conidia on the natural substrate were recorded shorter 15.8–20.9 × 6–8.3 μm (MFLUCC 18-0153, [2]) than conidia from the culture 22–27.5 × 6–7 μm (CBS 140002, this study) and 22–28 × 7–8 μm (NHL 2891 ex-type, [21]).

*Multiguttulispora dimorpha* produces brightly coloured pigments in vitro. On MLA, the species formed a conspicuous ochre to golden outer zone of submerged growth and yellow pigment diffused in the agar (Figure 9J). On OA, peach-pink pigment was formed in older colonies (Figure 9K). Distinct pigments produced in vitro have been reported by other authors. Toyazaki and Udagawa [21] observed deep yellow or deep orange pigment on OA. Crous et al. [76] described conspicuous red pigment (crystals) on OA.

*Multiguttulispora dimorpha* and *M. triseptata* are very similar; see the notes under the latter species for comparison.

**Figure 10:**
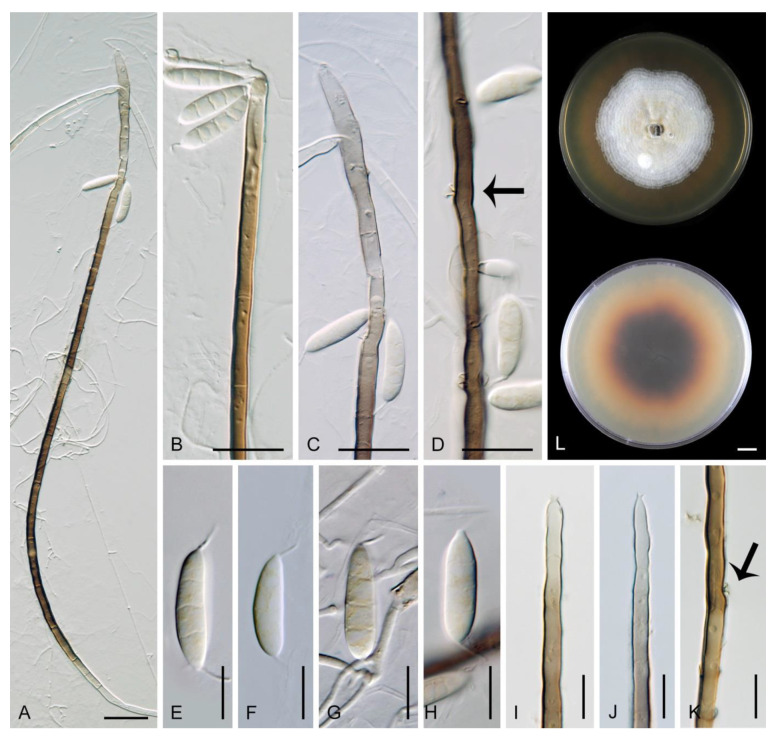
*Multiguttulispora triseptata*. (IMI 353690). (**A**) conidiophore (**B**–**D**,**K**) fertile parts of the conidiophores with lateral phialidic openings (arrows indicate collarettes) (**E**–**H**) conidia (**I**,**J**) tips of the conidiophores (**L**) colonies (front and reverse). Images: (**A**–**L**) on MLA after 8 weeks. Scale bars: (**A**) 25 μm; (**B**–**D**) 20 μm; (**E**–**K**) 10 μm; (**L**) 1 cm.

*Multiguttulispora triseptata* (Matsush.) Réblová & Hern.-Restr., comb. nov. MycoBank MB 839481 (Figure 10).

Basionym: *Codinaea triseptata* Matsush., Matsush. Mycol. Mem. 2: 4. 1981.

Synonym: *Dictyochaeta triseptata* (Matsush.) R.F. Castañeda, Fungi Cubense: 8. 1986.

Characteristics in culture: On CMD colonies 47–48 mm diameter, circular, flat, margin fimbriate, lanose, cobwebby at the margin, white with ochre tinge when aerial hyphae are sparse, with an orange-beige to olivaceous beige outer zone of submerged growth, pale ochre-pink pigment diffusing into the agar, reverse dark amber to dark brown. On MLA colonies 45–46 mm diameter, circular, flat with raised margin, margin entire to weakly fimbriate becoming curled, lanose, floccose, mucoid at the margin, finely furrowed, white with irregular yellow-orange patches, with an ochre to pale cinnamon outer zone of submerged growth, yellow-ochre pigment diffusing into the agar, reverse dark brown centrally, deep apricot towards the margin. On OA colonies 53–55 mm diameter, circular, flat, margin entire, velvety-lanose, white, olivaceous beige to olivaceous grey towards the periphery, white at the margin, locally with minute, ochre patches of aerial mycelium bearing ochre exudates, ochre to cinnamon pigment diffusing into the agar, reverse cinnamon to brown, ochre towards the margin. On PCA colonies 50–51 mm diameter, circular, flat, margin fimbriate, velvety, mucoid, smooth at the margin, white-grey, fawn towards the periphery ochre pigment diffusing into the agar, reverse dark brown. Sporulation was delayed (>8 weeks), moderate on MLA and PCA, absent on CMD and OA.

Colonies on PCA effuse, locally hairy, mycelium composed of branched, septate, hyaline hyphae 1.5–3 μm diameter. Anamorph: Setae absent. Conidiophores 260–823 μm long, 5–8.5 μm wide near the base, macronematous, single or arise in groups of 2–3 from knots of hyphal cells, erect, straight or flexuous, unbranched, occasionally branched in the apical part, septate, smooth, dark brown and thick-walled, paler and thinner-walled towards the apex. Conidiogenous cells 14–21.5 × 3–3.5(–5) μm, integrated, terminal, mono- and polyphialidic with 1–2 lateral openings while septa can be formed internally, single lateral phialidic apertures remain active along the upper part of the conidiophore, cylindrical, pale brown, subhyaline towards the apex; collarettes 1.5–3 μm wide, 1.5–2.5 μm deep, funnel-shaped, subhyaline, margin often curved downwards. Conidia 19–26 × (5–)5.5–6.5 μm (mean ± SD = 22.7 ± 1.9 × 5.8 ± 0.5 μm), ellipsoidal to oblong or ellipsoidal-fusiform, slightly curved, slightly tapering towards both ends, with a basal scar, 3-septate, hyaline, with straight or gently curved setula at each end, 3.5–6.5 μm long, positioned terminally at the apex and slightly subterminally at the base, conidia accumulate in slimy colourless fascicles. Synanamorph: not observed. Teleomorph: not observed.

Specimens examined: CUBA, San Antonio de los Baños, on unidentified fallen leaf, 2 February 1992, R.F. Castañeda-Ruíz (INIFAT C92/70, culture CBS 487.92). CUBA, on unidentified leaf, 1992, J. Guarro (culture IMI 353690).

Habitat and geographical distribution: Saprobe on dead leaves and decaying fruits or as a leaf endophyte of *Mucuna urens*, *Musa* sp. and other unidentified hosts, known in Cuba, Guyana, Peru, Pohnpei and USA ([22,74,75], this study).

Note: *Multiguttulispora triseptata* was described as *Codinaea triseptata* by Matsushima [22] from leaf litter in the USA. The type strain is not available for study (USA, Alabama, Tuskegee National Forest, on fallen leaves of a broadleaf tree, 23 June 1979, T. Matsushima, no. 9587). In the protologue, Matsushima [22] gave conidiophores 75–300 × 5–7 μm and conidia 21–30 × 6–7.5 μm in vitro. Castañeda-Ruíz [74] studied a Cuban collection on fallen leaves and described the conidiophores up to 450 μm long with conidia 24.5–37 × 7–8 μm under natural conditions. The two analysed strains from Cuba (CBS 487.92, IMI 353690) had conidia 19–26 × (5–)5.5–6.5 μm and conidiophores up to 823 μm, although shorter conidiophores up to 300 μm occurred frequently. A specimen of *Dictyochaeta triseptata* reported from Brazil by Cruz and Gusmão [77] belongs to a different species; it differs from *M. triseptata* in setae arranged with conidiophores in fascicles. *Multiguttulispora*, on the other hand, has solitary conidiophores that are sometimes arranged in small groups.

*Multiguttulispora triseptata* closely resembles *M. dimorpha* in conidial characteristics. Their conidial size overlaps considerably, which makes identification based on this sole character challenging. *Multiguttulispora triseptata* differs especially in mono- sometimes polyphialidic conidiogenous cells with only a few apertures and the absence of fertile zones of aggregated phialidic openings. Instead, single intercalary phialidic apertures occur along the upper part of the conidiophore.

**Figure 11:**
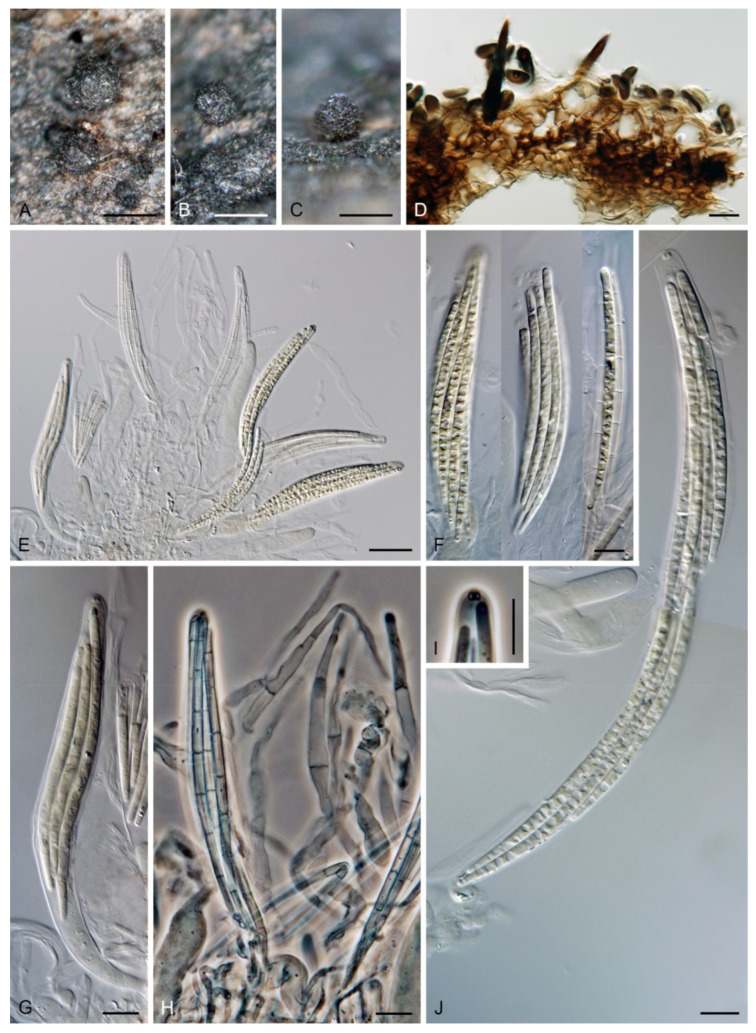
*Paragaeumannomyces hispidus* (PRM 900543 holotype). (**A**–**C**) ascomata (**D**) vertical section of the ascomal wall (**E**,**G**,**H**,**J**) asci and paraphyses (**F**) ascospores (**I**) ascal apex with an apical ring. Images: (**A**–**J**) from nature. Scale bars: (**A**–**C**) 250 μm; (**D**,**F**–**J**) 10 μm; (**E**) 25 μm.

*Paragaeumannomyces hispidus* (Réblová & Seifert) Réblová & Hern.-Restr., comb. nov. MycoBank MB 839482. (Figure 11).

Basionym: *Chaetosphaeria hispida* Réblová & Seifert, Sydowia 55: 323. 2003.

Description on natural substrate: Anamorph: not observed. Teleomorph: Ascomata 170–230 μm diam, 200–250 μm high, non-stromatic, superficial, solitary, conical, sub-globose to globose, papillate, dark brown, setose. Setae 27–37 μm long, (4.5–)5–6.5 μm wide near the base, acute, dark brown, opaque, unbranched, aseptate, never conidiogenous. Ostiole periphysate. Ascomatal wall fragile, 38–45 μm thick, three-layered; outer layer consisting of brown, globose to angular cells 9.5–14.5 μm diameter, middle layer consisting of dark brown, polyhedral to brick-like cells with opaque walls, inner layer consisting of several rows of thin-walled, compressed, subhyaline to hyaline cells. Paraphyses, septate, hyaline, slightly inflated, occasionally branched, 4–6(–7.5) μm wide near the base, tapering to 2.5–3 μm, longer than the asci. Asci 141–175 × 13–17 μm (mean ± SD = 153.0 ± 13.1 × 14.8 ± 1.3 µm), cylindrical-clavate, sessile or with a short stipe, apically narrowly rounded, ascal apex with a non-amyloid apical annulus 2–2.5 μm diameter, ca. 1.5 μm high, 8-spored. Ascospores 74–85(–87) × (3–)3.5–4 μm (mean ± SD = 79.7 ± 4.1 × 3.5 ± 0.4 µm), cylindrical to narrowly fusiform, straight, sigmoid or slightly curved, hyaline, 1–7-septate, not constricted at septa, tapering towards the base, rounded apically, smooth, 3–4-seriate, 4-seriate end-to-end or fasciculate in the ascus, with a negative dextrinoid reaction in Melzer’s reagent (partially adapted from Réblová and Seifert [19]).

Specimen examined: THAILAND, Nakhon Nayok Province, Khao Yai National Park, Bung Phai trail ca. 5 km NW from Khao Yai forest Headquarters on a way to Pak Chong, alt. 750 m, 14°28′N 10°23′E, on decaying wood, 6 September 2001, M. Réblová, G. J. Samuels & R. Nasit M.R. 2220/TH 511 (holotype of *Chaetosphaeria hispida* PRM 900543).

Habitat and geographical distribution: Saprobe on decaying wood, known only in Thailand.

Note: Re-examination of the holotype of *Ch. hispida* [19] revealed that the ascomatal wall is three-layered. The outer layer is partly deteriorated and it was initially overlooked. Based on the morphological study, this species is transferred to *Paragaeumannomyces*. The three-layered ascomatal wall with an outer layer composed of globose to angular cells is unique among species of the Chaetosphaeriaceae and is an important diagnostic trait in distinguishing between *Paragaeumannomyces* and *Ericiosphaeria*. *Paragaeumannomyces garethjonesii* [42] resembles *P. hispidus* in brown ascomata with short, acute spines and 7-septate ascospores, but differs in shorter asci (120–152 × 10.7–13.3 μm) and shorter ascospores (63.3–75 × 2.3–3.7 μm).

Catania et al. [78] described *Ch. hispida* var. *podocarpi* on bark and wood of *Podocarpus parlatorei* in Argentina. In morphological characteristics, it matches the genus *Ericiosphaeria* and is similar to *E. spinosa*, but differs in shorter ascospores (35.5–)40–49.5(–51) × 3–4 μm vs. 68–76 × 2–3 μm [3]. A dematiaceous hyphomycete growing in the juxtaposition to the ascomata was described as the anamorph. It has mononematous, simple, pigmented conidiophores with terminal conidiogenous cells and ellipsoidal-fusiform, 3-septate conidia with middle cells brown and end cells hyaline. The conidiogenous cells were depicted with several conidiogenous loci at the apex, but the mode of conidiogenesis was not described. The associated hyphomycete is reminiscent of *Cacumisporium capitulatum*, the anamorph of *Ch. decastyla* [79]. Interestingly, *C. capitulatum* is another member of the *Paragaeumannomyces* clade that includes teleomorphs with filiform to cylindrical, septate, asymmetrical ascospores. In *C. capitulatum*, the phialides extend above the collarette and have several subsequent narrow annellate proliferations; the conidia are formed successively on multiple conidiogenous loci and their arrangement at the tip of the phialide looks similar to that illustrated for the anamorph of *Ch. hispida* var. *podocarpi*. For now, we refrain from proposing a new combination at species rank for a taxon named as a variety of *Ch. hispida*. Its phylogenetic relationships should be resolved with molecular data.

*Phialogeniculata guadalcanalensis* Matsush., Bull. Nat. Sci. Mus. Tokyo 14: 472. 1971.

Synonyms: *Dictyochaeta guadalcanalensis* (Matsush.) Kuthub. & Nawawi, Mycol. Res. 95: 1220. 1991.

*Tainosphaeria obclavata* D.F. Bao, Z.L. Luo, K.D. Hyde & H.Y. Su, Fungal Divers. 99: 604. 2019.

Habitat and geographical distribution: Saprobe on decaying stems of *Musa paradisiaca* and wood of unidentified hosts in freshwater and terrestrial habitats. The species is known in Malaysia, Solomon Islands and Thailand [15,16,17,80].

Note: *Phialogeniculata* [16], typified with *P. guadalcanalensis*, was erected for saprobic dematiaceous hyphomycetes lacking setae and with macronematous, mononematous, erect, simple, bent to geniculate, dark brown conidiophores. The conidiogenous cells are polyphialidic, extending sympodially, with funnel-shaped collarettes. The conidia are obclavate, transversely septate, hyaline and without setulae. *Phialogeniculata guadalcanalensis* was described with conidiophores 35–70 × 4–6 μm, polyphialides with several lateral apertures surrounded by brown, stipitate, funnel-shaped collarettes and 1-septate, obclavate, hyaline conidia 18–27 × 4.5–5 μm [16,17]. *Tainosphaeria obclavata*, described from submerged wood in Thailand [15], matches the protologue of *P. guadalcanalensis* in all details. The holotype of *P. guadalcanalensis* (SOLOMON ISLANDS, Honiara, on wood of a broadleaf tree, 5 January 1970, T. Matsushima, MFC-2985) is not available for study. Based on original descriptions and illustrations of both species and their detailed morphological comparison, *T. obclavata* is treated as conspecific with *P. guadalcanalensis* and is reduced to synonymy.

To date, four species have been introduced in *Phialogeniculata*, namely *P. africana*, *P. dimorpha*, *P. guadalcanalensis* and *P. multiseptata* [16,17,65,81]. *Phialogeniculata guadalcanalensis* resembles *P. africana* [81], which differs in smaller obclavate conidia 11–16 × 2.5–3 μm, with the septum positioned close to the tapering apex.

*Phialoturbella* Réblová & Hern.-Restr., gen. nov. MycoBank MB 839483.

Etymology: *Phiale* (L), a broad, flat vessel referring to a phialidic conidiogenous cells, *turbella* (L), a little crowd, diminutive of *turba*, referring to crowded conidiophores.

Type species: *Phialoturbella lunata* (Z.L. Luo, K.D. Hyde & H.Y. Su) Réblová & Hern.-Restr.

Description: Colonies on the natural substrate effuse, hairy, mycelium partly superficial, partly immersed, hyphae brown, composed of conidiophores, occasionally ascomata. Anamorph: Setae absent. Conidiophores macronematous, mononematous, single or arise in groups from dark stromatic cells, unbranched, erect, straight or flexuous, septate, smooth, brown. Conidiogenous cells integrated, terminal, mono- or polyphialidic, extending percurrently and sympodially, cylindrical, pale brown, subhyaline towards the apex; collarettes funnel-shaped. Macroconidia falcate, lunate or oblong and curved, slightly truncate at the basal hilum, hyaline, aseptate, without setulae, accumulate in slimy fascicles. Microconidia (observed only in culture) falcate, lunate or oblong-clavate, curved, truncate at the basal hilum, hyaline, aseptate, without setulae, formed from the same conidiogenous loci. Teleomorph: Ascomata perithecial, non-stromatic, superficial, sub-globose to conical, papillate or with a beak-like neck, dark brown, glabrous. Ostiole periphysate. Ascomatal wall two-layered, carbonaceous. Paraphyses persistent, septate, hyaline. Asci unitunicate, cylindrical-clavate, sessile or with a short stipe, ascal apex with a non-amyloid apical annulus, 8-spored. Ascospores ellipsoidal, hyaline, aseptate, smooth.

Habitat and geographical distribution: Saprobes on decaying bark and wood, known in China and New Zealand ([2,15], this study).

Note: *Tainosphaeria* [3] grouped into three lineages in the ITS-28S phylogenetic tree (Figure 1). *Tainosphaeria crassiparies* and four other species clustered in a monophyletic clade and represent the core of the genus characterised by macronematous, solitary, simple conidiophores, usually monophialidic conidiogenous cells and falcate, setulate conidia. However, three *Tainosphaeria* species were not resolved congeneric with *T. crassiparies*. *Tainosphaeria aseptata*, *T. lunata*, and a morphologically similar strain ICMP 23826 from New Zealand, clustered as a separate lineage, introduced as the new genus *Phialoturbella* (*Ph.*). This is characterised by macronematous, solitary or crowded, simple conidiophores with mono- occasionally polyphialidic conidiogenous cells and aseptate, falcate to lunate conidia without setulae. *Tainosphaeria obclavata* is transferred to *Phialogeniculata*; for a discussion see above.

Several morphologically similar species of *Dictyochaeta* can be considered relatives or possible candidates for inclusion in *Phialoturbella*, namely *D. apiculata* [71], *D. botulispora* [9], *D. heteroderae* [82], *D. illinoensis* [83] and *D. occidentalis* [84]. Unfortunately, none of these species has available DNA sequences or cultures. Recollecting these taxa and obtaining axenic cultures and DNA data is necessary to resolve this little-known complex of species.

*Phialoturbella aseptata* (C.G. Lin & J.K. Liu) Réblová & Hern.-Restr., comb. nov. MycoBank MB 839484.

Basionym. *Tainosphaeria aseptata* C.G. Lin & J.K. Liu, Mycosphere 10: 683. 2019.

Habitat and geographical distribution: Saprobe on decaying wood, known only in China [2].

Note: For description and illustrations, refer to Lin et al. [2]. *Phialoturbella aseptata* has unbranched, solitary conidiophores, monophialidic conidiogenous cells extending percurrently and long fusiform, curved, aseptate conidia 14.4–18.9 × 3.3–4.4 μm [2]. On a photograph accompanying the protologue, a conidiophore is depicted with structures resembling persistent remnants of the collarettes, suggesting the conidiogenous cells can also form lateral openings and extend sympodially. The conidia are tapering apically and the basal end appears slightly truncate with an inconspicuous scar, which is visible on two images. Considering these characters, *D. illinoensis* [83] is highly similar to *P. aseptata* in features of conidia. The conidial size of both species overlaps, but the conidia of *D. illinoensis* are slightly longer in their upper range, 15.7–23.3 × 3.8–4.5 μm.

**Figure 12:**
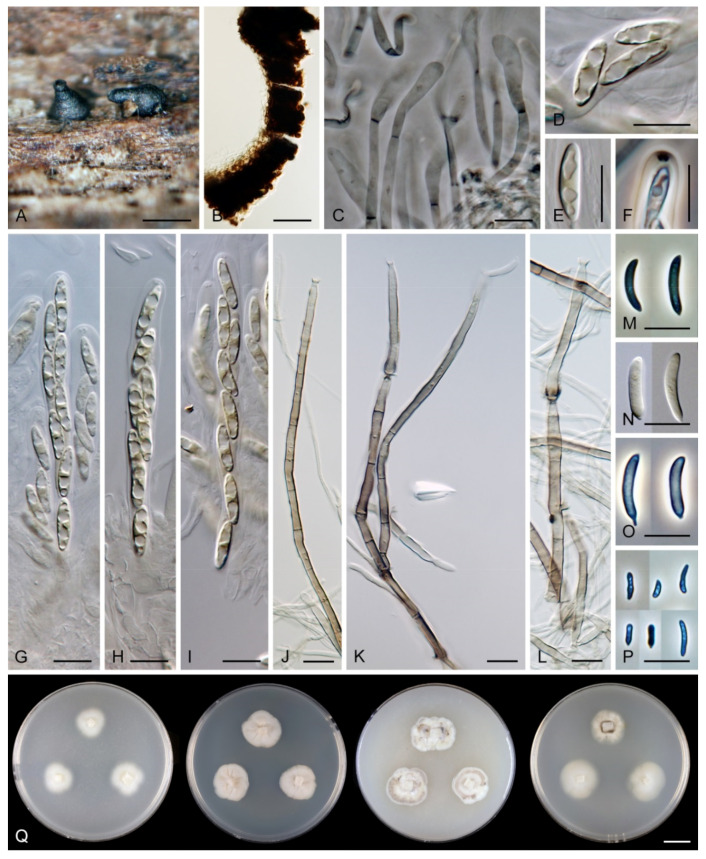
*Phialoturbella calva*. (ICMP 23826 ex-type) (**A**) ascomata (**B**) vertical section of the ascomal wall (**C**) paraphyses (**D**,**E**) ascospores (**F**) ascal apex with apical annulus (**G**–**I**) asci (**J**–**L**) conidiophores (**M**–**O**) macroconidia (**P**) microconidia (**Q**) colonies on CMD, MLA, OA and PCA after 4 weeks (from left to right). Images: (**A**–**I**) from nature; (**J**–**P**) on PCA after 6 weeks. Scale bars: (**A**) 250 μm; (**B**) 20 μm; (**C**–**P**) 10 μm; (**Q**) 1 cm.

*Phialoturbella calva* Réblová & Hern.-Restr., sp. nov. MycoBank MB 839563. (Figure 12).

Etymology: *Calvus* (L) naked, referring to the glabrous ascomata.

Type: NEW ZEALAND, Otago province, Clutha district, Catlins Coastal Rain Forest Park, MacLennan Range, Papatowai, Tautuku Nature Walk, on decaying bark of a trunk, 16 March 2005, M. Réblová M.R. 3265/NZ 525 (holotype PDD 119190 as dried culture, ex-type culture ICMP 23826).

Description on the natural substrate: Anamorph: not observed. Teleomorph: Ascomata 200–230 μm diam, 280–330 μm high, non-stromatic, superficial, solitary, conical, papillate or with a rostrate or beak-like neck, dark brown, glabrous, glossy. Ostiole periphysate. Ascomatal wall fragile, carbonaceous, 22–29 μm thick, two-layered; outer layer consisting of dark brown, polyhedral to brick-like cells with opaque walls, inner layer consisting of several rows of thin-walled, hyaline cells. Paraphyses septate, hyaline, more or less cylindrical, 2–3.5 μm wide, often widening upwards, apical cells clavate, 4–5 μm diameter, longer than the asci. Asci (64–)72–87 × 6.5–8.5 μm (mean ± SD = 79.9 ± 7.1 × 8.0 ± 0.6 µm), cylindrical-clavate, sessile or with a short stipe, apically rounded, ascal apex with a non-amyloid apical annulus 2–2.5 μm wide, 1.5–2 μm high. Ascospores 12–14.5 × 3–4 μm (mean ± SD = 13.2 ± 0.9 × 3.5 ± 0.4 µm), ellipsoidal, hyaline, aseptate, smooth, 2-seriate or obliquely uniseriate in the ascus.

Characteristics in culture: On CMD colonies 10–13 mm diameter, circular, flat, slightly convex centrally, margin entire, mucoid, isabelline at the centre, white towards the periphery, reverse isabelline. On MLA colonies 13–14 mm diameter, circular, convex, margin entire, mucoid-waxy, furrowed, white, later with irregular grey-brown spots due to pigmented mycelium and aggregated conidiophores, reverse isabelline. On OA colonies 13–15 mm diameter, circular, convex, margin entire, velvety to cobwebby, locally mucoid, white to isabelline, pale brown at the margin, with irregular dark brown spots due to aggregated conidiophores, reverse isabelline. On PCA colonies 13–15 mm diameter, circular, raised, margin entire, velvety-lanose, locally mucoid, white with irregular brown spots, especially at the centre, due to aggregated conidiophores, reverse white to isabelline. Sporulation was abundant on PCA and MLA, absent on CMD and OA.

Colonies on PCA effuse, mycelium composed of branched, septate, hyaline hyphae 1–2 μm diam. Anamorph: Setae absent. Conidiophores 102–300(–468) μm long, 2–3.5 μm wide above the base, tapering towards the base, unbranched or dichotomously branched in the lower part, erect, straight or flexuous, septate, smooth, pale brown to brown. Conidiogenous cells 21.5–46 × 2.5–3.5 μm, tapering to 1.5–2 μm below the collarette, integrated, terminal, mono- occasionally polyphialidic with one lateral opening, extending percurrently and sympodially, cylindrical, pale brown, subhyaline towards the apex; collarettes 2.5–3.5 μm wide, 1.5–2.5 μm deep, funnel-shaped, subhyaline, dark to pale brown when intercalary as remains after percurrent extension. Macroconidia 12.5–17 × 2–3 μm (mean ± SD = 14.5 ± 1.3 × 2.6 ± 0.2 μm), falcate to lunate or oblong and slightly curved, tapering towards both ends, with a basal hilum, hyaline, aseptate, without setulae, smooth, accumulate in slimy colourless fascicles. Microconidia 4–6.5 × 1–1.5 μm (mean ± SD = 5.0 ± 0.8 × 1.3 ± 0.2 μm), falcate or oblong-clavate, rounded at the apical end, tapering towards the base with a basal hilum, hyaline, aseptate, without setulae, formed from the same conidiogenous loci. Teleomorph: not observed.

Habitat and geographical distribution: Saprobe on decaying wood, known in New Zealand.

Note: In characters of conidia, phialides and conidiophores, *Ph. calva* closely resembles *Dictyochaeta apiculata* described from decaying wood in Japan [71], but the latter species differs by larger conidia, 20–30 × 4–5.5 μm.

*Phialoturbella lunata* (Z.L. Luo, K.D. Hyde & H.Y. Su) Réblová & Hern.-Restr., comb. nov. MycoBank MB 839485.

Basionym. *Tainosphaeria lunata* Z.L. Luo, K.D. Hyde & H.Y. Su, Fungal Diversity 99: 604. 2019.

Habitat and geographical distribution: Saprobe on submerged wood, known only in China [15].

Note: For description and illustrations, refer to Luo et al. [15]. *Phialoturbella lunata* has conidiophores 71–103 × 6–8 μm arising in groups from dark stromatic cells, terminating into a monophialide and fusiform, curved conidia 16–19 × 4.5–5.5 μm [15]. It resembles *Ph. aseptata* but differs in having conidiophores with a stromatic base and shorter and wider conidia.

**Figure 13:**
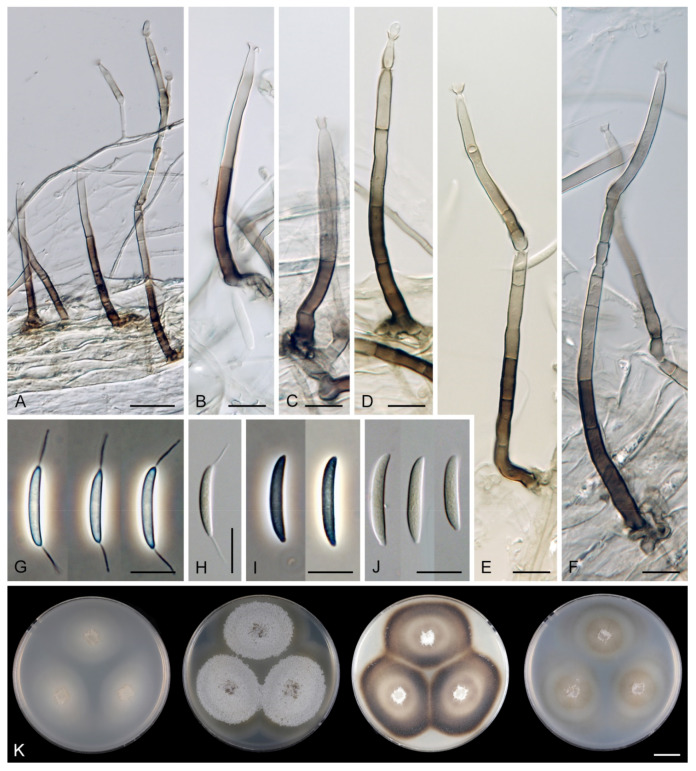
*Tainosphaeria cecropiae*. (CBS 101687 ex-type). (**A**–**F**) conidiophores (**G**–**J**) conidia (**K**) colonies on CMD, MLA, OA and PCA after 4 weeks (from left to right). Images: (**A**–**J**) on CMA with *Urtica* stems after 8 weeks. Scale bars: (**A**) 20 μm; (**B**–**J**) 10 μm; (**K**) 1 cm.

*Tainosphaeria cecropiae* Réblová & Hern.-Restr., sp. nov. MycoBank MB 839486 (Figure 13).

Etymology: named after the host plant, *Cecropia*.

Type: PUERTO RICO, Luquillo National Forest, El Toro Trail, on decaying petiole of *Cecropia* sp., June 1998, W. Gams (holotype CBS H-24745 as dried culture, ex-type culture CBS 101687).

Characteristics in culture: On CMD colonies 28–32 mm diameter, circular, flat, margin entire, funiculose at the inoculation block, mucoid towards the margin, white to isabelline, reverse white. On MLA colonies 60–62 mm diameter, circular, convex, margin fimbriate, funiculose and partially mucoid at the centre of the colony, velvety, floccose, appearing powdery, mucoid at the margin, white-beige, olivaceous grey-brown at the margin, reverse grey-brown. On OA colonies 61–63 mm diameter, circular, flat, margin entire, lanose, funiculose at the inoculation block becoming mucoid, zonate, white centrally, beige-brown with a dark brown outer zone, reverse of the same colours. On PCA colonies 65–67 mm diameter, circular, flat, margin entire, mucoid, funiculose at the inoculation block, zonate, white centrally, pale brown, beige towards the periphery, reverse of the same colours. Sporulation was absent on all media.

Colonies on CMA with *U. dioica* stems effuse, mycelium composed of branched, septate, subhyaline to pale brown hyphae 1–2.5 μm diam. Anamorph: Setae absent. Conidiophores 40–130(–150) μm long, 3.5–4.5 μm wide above the base, macronematous, mononematous, unbranched, erect, straight or flexuous, septate, smooth, dark brown and thick-walled in the lower part becoming pale brown to subhyaline and thinner-walled towards the apex, usually the first 2–3 cells above the base are darker than the rest of the conidiophore. Conidiogenous cells 15–39 × 3.5–4 μm, tapering to 1.5(–2) μm below the collarette, integrated, terminal, mono- rarely polyphialidic with one lateral opening, extending percurrently and sympodially, cylindrical, pale brown, subhyaline towards the apex; collarettes 3–4 μm wide, 1.5–2.5 μm deep, funnel-shaped, pale brown to subhyaline. Conidia falcate, tapering towards both ends, slightly truncate at the basal hilum, hyaline, aseptate, smooth. Two kinds of conidia are produced from the same conidiogenous loci: conidia without setulae 15.5–21 × 2.5–3.5 μm (mean ± SD = 18.8 ± 1.5 × 3.0 ± 0.3 μm), conidia with setulae 16–19 × 2.5–3 μm (mean ± SD = 17.5 ± 0.8 × 2.7 ± 0.2 μm), setulae straight or gently curved 6.5–8.5 μm long, conidia accumulate in slimy colourless fascicles. Teleomorph: not observed.

Habitat and geographical distribution: Saprobe on decaying petioles of *Cecropia* sp., known only in Puerto Rico.

Note: This species sporulated only on stems of *U. dioica* on CMA. When grown in culture, *T. cecropiae* formed two kinds of conidia, with and without setulae, of which the latter were more abundant. Although two synanamorphs with conidia without setulae were reported for *T. crassiparies* [3], they differed in shape and size from the falcate, setulate conidia of the anamorph. The size of setulate and non-setulate conidia of *T. cecropiae* overlaps considerably, suggesting that instead of the presence of a synanamorph, the setulae formation in some conidia of *T. cecropiae* may be delayed or the setulae easily detach. The conidiophores have the first 2–3 cells above the base noticeably darker than the rest of the conidiophore, giving them an almost bicolour appearance. Except for *T. crassiparies*, species of *Tainosphaeria* share similar conidial size (13.5–19 × 2–3.5 μm) and therefore, it is difficult to distinguish them based on this character alone. *Tainosphaeria cecropiae* differs from other members of the genus by characteristically pigmented conidiophores and formation of setulate and non-setulate conidia in culture.

The strain of *T. cecropiae* was originally deposited under the name *Codinaea coffeae* [85]. The latter species was isolated from nursery soil of *Coffea arabica* in Mexico and differs from *T. cecropiae* in shorter conidia (10.8–18 × 3.4–5 μm) and brown conidiophores becoming paler towards the apex without distinct dark and pale zones.

## 4. Discussion

New lines of evidence, based on results of phylogenetic analysis of the ITS-28S DNA sequences and comparative morphological studies of newly collected material, ex-type strains and other isolates, provided a sound basis for assessing generic concepts of *Menisporopsis*, *Multiguttulispora* and *Tainosphaeria*. Five species of *Menisporopsis*, representing the core of the genus, clustered in a monophyletic clade with *M. theobromae*. *Menisporopsis* has hyaline, aseptate conidia with one or more setulae inserted at each end or irregularly and synnemata surrounding the central, dark brown seta. The unique combination of these morphological traits makes *Menisporopsis* a well-recognisable genus. Our analysis confirms that the interspecific variation is based mainly on conidial shape and characters of setulae, i.e., their number and position on conidia. Identification key and synopsis of known species of *Menisporopsis* were published in Tsui et al. [5], Castañeda-Ruíz et al. [6] and Cruz et al. [70]. *Menisporopsis novae-zelandiae* and other isolates with 1-septate, setulate conidia and synnemata that grow unilaterally along the seta were resolved as a separate, strongly supported clade. They are accommodated in the new segregate genus *Arcuatospora* in this study. *Menisporopsis ludoviciana* [10,11] is another species that does not conform to the generic description. Castañeda-Ruíz et al. [13] transferred *M. ludoviciana* to *Vermiculariopsiella* (Vermiculariopsiellales) based on original description and illustration. However, its systematic placement awaits verification with molecular data.

The interspecific variability of *Multiguttulispora* was assessed using novel ITS and 28S rDNA sequences and a comparison of morphological data. Two species initially accommodated in *Codinaea*, *C. dimorpha* and *C. triseptata* [21,22], were resolved as being congeneric with the type species *M. sympodialis*, which is treated as a synonym of *M. dimorpha*. The conidial shape and septation, the extension of the phialides, and the arrangement of the collarettes along the robust, dark brown, almost opaque setiform conidiophores are the most valuable diagnostic features that distinguish *Multiguttulispora* from *Codinaea*. The latter genus is delimited to species with setae and conidiophores arising in tufts with mono- or polyphialidic conidiogenous cells and hyaline, usually falcate, aseptate conidia with setulae [14]. In the phylogenetic tree, these genera formed distantly related lineages.

The *Tainosphaeria* complex has been revised. Based on anamorphic characteristics, species previously classified in *Tainosphaeria* can be distinguished into three morphological groups that are consistent with revealed phylogenetic relationships (Figure 1). These lineages include *Tainosphaeria*, *Phialogeniculata* and *Phialoturbella*. They differ mainly in the characteristics of conidia and conidiophores. *Tainosphaeria* contains five species, i.e., *T. cecropiae*, *T. crassiparies*, *T. jonesii*, *T. monophialidica*, and *T. siamensis*, and is delimited to fungi with mononematous, pigmented, simple conidiophores, mono- occasionally polyphialidic conidiogenous cells and falcate, hyaline, aseptate, conidia with setulae. The new segregate genus *Phialoturbella* contains two former *Tainosphaeria* species, such as *Ph. aseptata*, *Ph. lunata* and the new species *Ph. calva*. Their conidia are similar to those of *Tainosphaeria,* but lack setulae. *Tainosphaeria obclavata* is found conspecific with *Phialogeniculata guadalcanalensis* and is transferred to synonymy. *Phialogeniculata* differs from *Phialoturbella* and *Tainosphaeria* in septate, asymmetrical conidia and geniculate conidiophores with funnel-shaped and slightly stipitate collarettes, and accommodates four species. Although Kuthubutheen and Nawawi [80] transferred *P. guadalcanalensis* to *Dictyochaeta*, such a relationship could not be confirmed using molecular DNA data. *Dictyochaeta* is resolved as a well-supported lineage and delimited to species with setae accompanied by shorter conidiophores with mono- or polyphialidic conidiogenous cells and falcate to falcate-clavate, slightly asymmetrical conidia without septa and setulae [32,86].

In the phylogenetic tree based on the combined ITS-28S sequences, two other morphotypes were revealed in the *Tainosphaeria* clade. The genus *Anacacumisporium* with a single species, *A. appendiculatum*, was described for saprobic lignicolous fungi in China [18]. It can be recognised in having fusiform, septate conidia with middle cells brown and end cells hyaline, and a simple setula at each end, borne on terminal mono- or polyphialidic conidiogenous cells. *Flectospora* represents the fifth lineage with distinct morphology in the *Tainosphaeria* clade. It includes species with hyaline, aseptate, ellipsoidal to obovoid, slightly curved conidia borne on monophialides on pigmented conidiophores, superficial dark ascomata, short-stipitate asci with hyaline, ellipsoidal, septate ascospores. One of the species, *F. laminata*, is introduced for the alleged chloridium-like anamorph of *Ch. hispida* [19]. Revision of the holotype of *Ch. hispida* and phylogenetic analysis of members of the Chaetosphaeriaceae led to a new taxonomic treatment of *Ch. hispida*, morphologically similar to *Ch. spinosa*, and clarification of the anamorph of *Ch. hispida*. New combinations are proposed for both *Chaetosphaeria* species. *Chaetosphaeria hispida* is transferred to *Paragaeumannomyces* and *Ch. spinosa* is accepted in *Ericiosphaeria*. *Chaetosphaeria* s. str., represented by the type species *Ch. innumera* in the phylogenetic analysis, forms a separate lineage (Figure 1).

Although species of *Chaetosphaeria* have hyaline, transversely septate ascospores that are primarily symmetrical, ellipsoidal or fusiform, a handful of species have cylindrical, cylindrical-fusiform to filiform, sometimes asymmetrical ascospores. Based on the results of ITS-28S phylogenetic analysis, species with cylindrical-filiform and asymmetrical ascospores grouped in one clade only. Apart from *Ericiosphaeria*, this clade also includes *Paragaeumannomyces* and other species such as *Cacumisporium capitulatum*, *Catenularia cubensis*, *Ch. fusiformis*, *Ch. lignomollis*, *Exserticlava vasiformis* and *Stanjehughesia hormiscioides* [3,7,20,79,87,88]. Of these taxa, *Cacumisporium*, *Ericiosphaeria* and *Paragaeumannomyces* possess asymmetrical ascospores. The similarity between *Ericiosphaeria* and *Paragaeumannomyces* in the ascospore and ascoma characteristics is undisputable; they differ mainly in the anatomy of the ascomatal wall. In the phylogenetic analysis, they are shown as sister taxa, although their relationship is statistically supported only in the ML analysis. A recent account of the morphology, taxonomy and phylogeny of *Paragaeumannomyces* has been published in Réblová et al. [20]. The authors [20] reported a correlation between ascoma morphology and ascospore reaction in Melzer’s reagent. The ascospores of species of *Paragaeumannomyces* with glabrous ascomata, occasionally with ostiolar setae, exhibit a strong dextrinoid reaction in Melzer’s reagent, while ascospores of species with setose ascomata have a negative or weak reaction. Our observations of *P. hispidus* are in agreement with Réblová et al. [20]; the ascomata are covered with acute setae over the whole surface and ascospores do not stain in Melzer’s reagent. Taxonomy, systematics and relationships of *Ch. fusiformis* and *Ch. lignomollis* are more complex and beyond the scope of this study (Réblová et al., unpubl.).

In this study, we contributed to the delimitation of several monophyletic chaetosphaeriaceous genera and identification of unique sets of morphological characters that define them and are phylogenetically consistent. Our study provided new data and revealed new phylogenetic relationships in the Chaetosphaeriaceae.

## Figures and Tables

**Table 1:** Taxa, isolate information and GenBank accession numbers for sequences. New sequences determined for this study and taxonomic novelties are given in bold.

Taxon	Strain	Status ^1^	Country	Host	Substrate	GenBank Accessions	
						ITS	28S
*Arcuatospora novae-zelandiae*	CBS 109474		Venezuela	*Nectandra* sp.	decaying leaf	MW984569	MW984552
*A. novae-zelandiae*	CBS 109476		Venezuela	*Nectandra* sp.	decaying leaf	MW984570	MW984553
*A. seorsa*	CBS 147509		Thailand	*Dipterocarpus* sp.	decaying pod	MW984571	MW984554
*A. seorsa*	CBS 147510	T	Thailand	broad leaf tree	decaying leaf	MW984572	MW984555
*Arcuatospora* sp. 1	CBS 694.74		Japan	unidentified	decaying leaf	MW984573	MW984556
*Arcuatospora* sp. 2	MUCL 43189		Nepal	unidentified	unknown	MW984574	MW984557
*Ericiosphaeria spinosa*	S.M.H. 2754	T	USA	*Betula* sp.	decaying bark	MW984575	AF466079
*Flectospora laminata*	CBS 112964	T	Thailand	unidentified	decaying wood	MW984576	MW984558
*Flectospora* sp.	ICMP 23840		New Zealand	unidentified	decaying wood	MW984577	MW984559
*Menispora caesia*	CBS 144659		Czech Republic	*Quercus* sp.	decaying wood	MW984578	MW984560
*Menisporopsis pirozynskii*	MUCL 47217		Congo	unidentified	decaying leaf	MW984579	MW984561
*M. theobromae*	MUCL 41079		Venezuela	unidentified	dead leaf	MW984580	MW984562
*M. theobromae*	MUCL 40984		Venezuela	unidentified	decaying leaf	MW984581	MW984563
*Multiguttulispora dimorpha*	CBS 140002		Malaysia	*Eucalyptus* sp.	twig	MW984582	MW984564
*M. triseptata*	CBS 487.92		Cuba	unidentified	fallen leaf	MW984583	MW984565
*M. triseptata*	IMI 353690		Cuba	unidentified	leaf	MW984584	MW984566
*Phialoturbella calva*	ICMP 23826	T	New Zealand	unidentified	decaying bark	MW984585	MW984567
*Tainosphaeria cecropiae*	CBS 101687		Puerto Rico	*Cecropia* sp.	decaying petiole	MW984586	MW984568
*Tainosphaeria crassiparies*	S.M.H. 1934	T	Puerto Rico	*Hymenaea* sp.	seed pod	MW984587	AF466089

^1^ Note: T denotes ex-type strains.

## Data Availability

All sequences generated in this study were submitted to GenBank (ITS: MW984569–MW984587; 28S: MW984552–MW984568).

## Supplementary Materials

The following are available online at https://www.mdpi.com/article/10.3390/jof7060438/s1, Table S1: Taxa, isolate information and accession numbers for sequences retrieved from GenBank.
