# Supplementary material for: Reflections on Menisporopsis, Multiguttulispora and Tainosphaeria Using Molecular and Morphological Data
Source: J Fungi (Basel). 2021 May 31;7(6):438. doi: 10.3390/jof7060438 (PMC8227749; doi:10.3390/jof7060438)
Supplement: Supplementary file 1 [file jof-07-00438-s001.zip › jof-1220375-supplementary.pdf]

Table S1. Taxa, isolate information and accession numbers for sequences retrieved from GenBank.

Taxon	Strain	Status	Country	Host	Substrate	GenBank accessions	
						ITS	28S
Adautomilanezia caesalpiniae	CC-LAMIC 102/12	T	Brazil	*Caesalpina echinata*	wood	KX821777	KU170671
Achrochaeta talbotii	ICMP 15161		New Zealand	unidentified	decaying wood	MT454480	MT454495
Anacacumisporium appendiculatum	HMAS 245593	T	China	broad-leaved tree	dead stems	KP347129	KT001553
Anacacumisporium appendiculatum	HMAS 245602		n/a	n/a	n/a	KT001556	KT001554
Brunneodinemasporium brasiliense	CBS 112007	T	Brazil	unidentified	decaying leaf	JQ889272	JQ889288
Brunneodinemasporium jonesii	GZCC 16-0050	T	China	unidentified	decaying wood	KY026058	KY026055
Cacumisporium capitulatum	FMR 11339		Spain	unidentified	decaying wood	HF677176	HF677190
Calvolachnella guaviyunis	CBS 134695	T	Uruguay	*Myrcianthes pungens*	bark	KJ834524	KJ834525
Catenularia cubensis	S.M.H. 3258		Costa Rica	unidentified	decaying wood	—	AF466067
Chaetosphaeria fusiformis	CBS 101429		Czech Republic	*Abies alba*	decaying bark	AF178554	AF178554
Chaetosphaeria innumera	M.R. 1175		Czech Republic	*Fagus sylvatica*	decaying wood	AF178551	AF178551
Chaetosphaeria lignomollis	S.M.H. 3015	T	Puerto Rico	unidentified	decaying wood	EU037896	AF466073
Chaetosphaeria myriocarpa	CBS 264.76	N	The Netherlands	unidentified	decaying wood	AF178552	AF178552
Chaetosphaeria pygmaea	M.R. 1365		Czech Republic	*Fagus sylvatica*	decaying wood	AF178545	AF178545
Chloridium caesium	CBS 102339		Austria	*Salix cinerea*	decaying wood	AF178564	AF178564
Chloridium gonytrichii	CBS 195.60		South Africa	unidentified	unknown	MH857954	MH869503
Chloridium virescens	CBS 152.53		France	*Acer* sp.	unknown	MH857142	MH868678
Codinaea paniculata	CBS 145098	T	France	unidentified	submerged wood	MT118230	MT118201
Codinaeopsis gonytrichodes	CBS 593.93		Japan	unidentified	decaying plant material	AF178556	AF178556
Conicomyces pseudotransvaalensis	HHUF 29956	T	Japan	*Machilus japonica*	dead twig	LC001710	LC001708
Cryptophiale hamulata	MFLUCC 18-0098	E	Thailand	unidentified	decaying leaf	—	MG386756
Cryptophiale udagawae	GZCC 18-0047		China	unidentified	decaying wood	MN104608	MN104619
Cryptophialoidea fasciculata	MFLU 18-1499		Thailand	unidentified	submerged wood	MH758195	MH758208
Dendrophoma cytisporoides	CBS 144107		Germany	*Buxus sempervivens*	decaying bark	MT118234	MT118205
Dictyochaeta callimorpha	ICMP 15155		New Zealand	unidentified	decaying wood	MT454484	MT454499
Dictyochaeta fuegiana	ICMP 15153	T	New Zealand	unidentified	decaying wood	MT454487	EF063574
Dictyochaeta querna	CBS 145503		Czech Republic	*Quercus cerris*	acorn	MT454489	MT454503
Dictyochaeta siamensis	MFLUCC 15-0614	T	Thailand	unidentified	submerged decaying twig	KX609955	KX609952
Dictyochaeta terminalis	GZCC 18-0085	T	China	unidentified	decaying leaves	MN104613	MN104624
Dinemasporium cruciferum	HHUF 30001		Japan	*Arundo donax*	unknown	AB900895	AB934039
Dinemasporium decipiens	CBS 592.73		Suriname	n/a	soil under *Elaeis guineensis*	JQ889275	JQ889291

Dinemasporium pseudoindicum	CBS 127402	T	USA	n/a	soil of tallgrass prairie	JQ889277	JQ889293
Ellisembia aurea	CBS 144403	T	France	*Sambucus nigra*	decaying wood	MH836375	MH836376
Ellisembia folliculata	CBS 101317		France	*Salix* sp.	decaying wood	—	AF261071
Eucalyptostroma eucalypti	CBS 142074	T	Malaysia	*Eucalyptus pellita*	leaf spots	KY173408	KY173500
Exserticlava vasiformis	TAMA 450		Japan	unidentified	plant debris	—	AB753846
Infundibulomyces cupulatus	BCC 11929	T	Thailand	*Lagerstroemia* sp.	dead leaf	EF113976	EF113979
Infundibulomyces oblongisporus	BCC 13400	T	Thailand	unidentified, angiosperm	leaf litter	EF113977	EF113980
Kionochaeta castaneae	GZCC 18-0025	T	China	*Castanea mollissima*	decaying seed shell	MN104610	MN104621
Kionochaeta microspora	GZCC 18-0036	T	China	unidentified	decaying wood	MN104607	MN104618
Kionochaeta ramifera	MUCL 39164		Cuba	unidentified	leaf	MW144421	MW144404
Menispora ciliata	CBS 122131	T	Czech Republic	*Acer campestre*	decaying wood	EU488736	MH874726
Menispora tortuosa	DAOM 231154		unknown	unidentified	unknown	KT225527	AY544682
Menisporopsis anisospora	CBS 109475	T	Venezuela	*Wettinia praemorsa*	decaying petiole	MH862827	MH874421
Menisporopsis breviseta	GZCC 18-0071	T	China	unknown	decaying leaves	MN104612	MN104623
Menisporopsis dushanensis	GZCC 18-0084	T	China	unidentified	decaying leaves	MN104615	MN104626
Menisporopsis theobromae	MFLUCC 15-0055		Thailand	unidentified	submerged decaying wood	KX609957	KX609954
Multiguttulispora dimorpha	MFLUCC 18-0153	T	Thailand	unidentified	decaying plant	MN104606	MN104617
Nawawia filiformis	MFLUCC 17-2394		Thailand	unidentified	decaying wood	MH758196	MH758209
Neopseudolachnella acutispora	MAFF 244358	T	Japan	*Pleioblastus chino*	dead twigs	AB934065	AB934041
Neopseudolachnella magnispora	MAFF 244359	T	Japan	*Sasa kurilensis*	dead twigs	AB934066	AB934042
Paliphora intermedia	CBS 896.97	I	Australia	unidentified	leaf litter	MH862682	EF204501
Paragaeumannomyces albidus	PDD 92537	T	New Zealand	*Nothofagus* sp.	decaying wood	EU037890	EU037898
Paragaeumannomyces bombycinus	PDD 92538	T	New Zealand	*Nothofagus* sp.	decaying wood	EU037892	—
Paragaeumannomyces garethjonesii	MFLUCC 15-1012	T	Thailand	Fabaceae	seed pod	KY212751	KY212759
Paragaeumannomyces longisporus	ILLS00121385		USA	unidentified	decaying wood	MT118237	MT118211
Paragaeumannomyces panamensis	S.M.H. 3596	T	Panama	unidentified	decaying wood	AY906948	MT118218
Paragaeumannomyces raciborskii	S.M.H. 3119		Puerto Rico	unidentified	decaying wood	AY906953	AY436402
Paragaeumannomyces rubicundus	S.M.H. 3221	T	Costa Rica	unidentified	decaying wood	MT118242	MT118224
Phaeostalagmus cyclosporus	CBS 663.70		The Netherlands	*Quercus* sp.	decaying bark	MH859892	MH871680
Phialogeniculata guadalcanalensis *	MFLUCC 18-0260	T	Thailand	unidentified	decaying wood	MK828625	MK835825
Phialosporostilbe scutiformis	MFLUCC 17-0227	T	China	unidentified	submerged decaying wood	MH758194	MH758207
Phialoturbella aseptata	GZCC 18-0044	T	China	unidentified	decaying wood	MN104611	MN104622
Phialoturbella lunata	MFLUCC 18-0642	T	China	unidentified	submerged wood	MK828624	MK835824
Polynema podocarpi	CBS 144415	T	New Zealand	*Podocarpus totara*	unknown	MH327797	MH327833
Pseudodinemasporium fabiforme	CBS 140010		Malaysia	*Acacia mangium*	leaf spots	KR611889	KR611906

Pseudolachnea fraxini	CBS 113701	T	Sweden	*Fraxinus excelsior*	unknown	JQ889287	JQ889301
Pseudolachnea hispidula	MAFF 244365		Japan	*Morus bombycis*	dead twig	AB934072	AB934048
Pseudolachnella asymmetrica	MAFF 244366	T	Japan	*Phyllostachys nigra* var. h	dead twig	AB934073	AB934049
Pseudolachnella scolecospora	MAFF 244379		Japan	Sasa sp.	dead twigs	AB934086	AB934062
Pyrigemmula aurantiaca	CBS 126743	T	Hungary	*Vitis vinifera*	bark	HM241692	HM241692
Rattania setulifera	GUFCC 15-501	T	India	*Calamus thwaitesii*	leaves	GU191794	HM171322
Sporoschisma longicatenatum	MFLUCC 16-0180	T	Thailand	unidentified	submerged decaying wood	KX505871	KX358077
Sporoschisma mirabile	FMR 11247		Spain	unidentified	dead wood	HF677174	HF677183
Sporoschisma taitense	KUMCC 15-0241		China	unidentified	submerged wood	KX455865	KX455858
Stanjehughesia hormiscioides	CBS 102664		Ukraine	*Fagus sylvatica*	decaying wood	—	AF261069
Striatosphaeria castanea	CBS 145352	T	French Guinea	woody liana	decaying periderm	MT118244	MT118229
Striatosphaeria codinaeophora	M.R. 1230		Puerto Rico	*Dacryodes excelsa*	decaying wood	AF178546	AF178546
Tainosphaeria jonesii	GZCC 16-0065	P	China	unidentified	submerged decaying wood	KY026060	KY026057
Tainosphaeria jonesii	GZCC 16-0053		China	unidentified	decaying wood	KY026059	KY026056
Tainosphaeria monophialidica	MFLUCC 18-0146	T	Thailand	unidentified	decaying wood	—	MN104616
Tainosphaeria siamensis	MFLUCC 15-0607	T	Thailand	unidentified	submerged decaying wood	KX609956	KX609953
Thozetella fabacearum	MFLUCC 15-1020	T	Thailand	Fabaceae	seed pod	KY212754	KY212762
Thozetella nivea	n/a		unknown	unidentified	unknown	EU825201	EU825200
Thozetella tocklaiensis	CBS 378.58	T	India	*Camellia sinensis*	decaying flower	MH857817	MH869349
Tracylla aristata	CBS 141404	E	Australia	*Eucalyptus regnans*	leaf	KX306770	KX306795
Tracylla eucalypti	CBS 144429	T	Colombia	*Eucalyptus urophylla*	spots on living leaves	MH327810	MH327846
Zanclospora iberica	CBS 130426	T	Spain	unidentified	decaying wood	KY853480	KY853544
Zanclospora novae-zelandiae	ICMP 15781	E	New Zealand	*Fuscospora cliffortioides*	decaying wood	MW144429	MW144411
Zanclospora xylophila	ICMP 22737	T	New Zealand	unidentified	decaying wood	MW144437	MW144417

Notes

T, E, I and P denote ex-type, ex-epitype, ex-isotype and ex-paratype strains.

* ex-type of *Tainosphaeria obclavata*
